# Comprehensive transcriptome, miRNA and kinome profiling identifies new treatment options for personalized lung cancer therapy

**DOI:** 10.1002/ctm2.70177

**Published:** 2025-02-24

**Authors:** Shen Zhong, Yvonne Börgeling, Patrick Zardo, Danny Jonigk, Jürgen Borlak

**Affiliations:** ^1^ Centre for Pharmacology and Toxicology Hannover Medical School Hannover Germany; ^2^ Institute of Virology University of Münster Münster Germany; ^3^ Clinic for Cardiothoracic and Transplantation Surgery Hannover Medical School Hannover Germany; ^4^ Institute for Pathology Hannover Medical School Hannover Germany

**Keywords:** kinase inhibitor, lung cancer, neoadjuvant therapy, precision medicine

## Abstract

**Background:**

Basic research identified oncogenic driver mutations in lung cancer (LC). However, <10% of patients carry driver mutations. Thus, most patients are not recommended for first‐line kinase inhibitor (KI)–based therapies. Through enabling technologies and bioinformatics, we gained deep insight into patient‐specific signalling networks which permitted novel KI‐based treatment options in LC.

**Methods:**

We performed molecular pathology, transcriptomics and miRNA profiling across 95 well‐characterized LC patients. We confirmed results based on cross‐linked immunoprecipitation‐sequencing data, and used *N* = 524 adeno‐ and 497 squamous cell carcinomas as validation sets. We employed the PamGene platform to identify aberrant kinases, validated the results by evaluating independent siRNA and CRISPR‐mediated mRNA knockdown studies in human LC cell lines.

**Results:**

Transcriptomics revealed 439, 1240, 383 and 320 significantly upregulated genes, respectively, for adeno‐, squamous, neuroendocrine and metastatic cases, and there are 1092, 1477, 609 and 1267 downregulated DEGs. Based on gene enrichment analysis and experimentally validated miRNA–gene interactions, we constructed regulatory networks specific for adeno‐, squamous, neuroendocrine and metastatic LC. Molecular profiling discovered 137 significantly upregulated kinases (range 2–26‐fold) of which 65 and 72, respectively, are tyrosine and serine‐threonine kinases while 6 kinases carry driver mutations. Meanwhile, there are 21 kinases commonly upregulated irrespective of the histological type of LC. Bioinformatics decoded networks in which kinases function as master regulators. Typically, the networks consisted of 14, 9, 16 and 19 highly regulated kinases in adeno‐, squamous, neuroendocrine and metastatic LC. Inhibition of kinases which function as master regulators disrupted the signalling networks, and their gene knock‐down studies confirmed inhibition of cell proliferation in a panel of human LC cell lines. Additionally, the proposed molecular profiling enables KI‐based therapies in patients with acquired drug resistance.

**Conclusions:**

Our study broadens the perspective of KI‐based therapies in LC, and we propose a framework to overcome acquired drug resistance.

## Graphical abstract

Kinase inhibition–based therapies are recommended for a small subset of LC patients with driver mutations. Through enabling technologies and bioinformatics, we demonstrate the potential of KI‐based therapies based on the patient specific needs. Thus, most LC patients become eligible for such treatment algorithms.

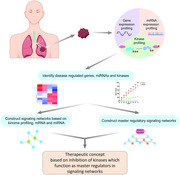

## 1 BACKGROUND

Lung cancer (LC) is the second most frequently diagnosed cancer worldwide and despite significant progress in its treatment, LC remains the primary cause of cancer death. By large, LC is a preventable disease, and according to Cancer Research UK, tobacco product use, occupation and air pollution accounts for 72%, 13% and 8%, respectively, of the disease burden (https://www.cancerresearchuk.org/health‐professional/cancer‐statistics/statistics‐by‐cancer‐type/lung‐cancer/risk‐factors). Furthermore, about 70% of LC patients are diagnosed at advanced‐stages of disease.^1^

Over the last decade, an amazing progress in LC therapy has been achieved with significant reductions in surgical complications due to minimal invasive approaches, that is, video‐assisted thoracoscopic surgery over open thoracotomy, improvements in radiotherapy and molecular testing and new concepts for systemic and targeted therapy. Nowadays, immune checkpoint inhibitors (ICIs) and chemotherapeutics are the mainstay of therapy, while patients with driver mutations are given targeted therapies.^2^, ^3^

In general, LC is divided into small (SCLC) and non–small cell carcinoma (NSCLC), and the latter group accounts for about 80%–85%. NSCLC is further divided into adeno‐ (AD), squamous cell (SQ) and other histological subtypes such as large‐cell carcinoma,^4^ and AD patients may harbour common oncogenic driver alterations, that is, Kirsten rat sarcoma virus (KRAS, about 30%), epidermal growth factor receptor (EGFR) (14%–30%), BRAF (5%–7%), anaplastic lymphoma receptor (ALK) (1%–5%), MET Proto‐Oncogene, Receptor Tyrosine Kinase (METs) (1%–3%) and ERBB2 (2%–6%).^5^, ^6^ Moreover, among SQ patients, genetic alterations have been reported for phosphatidylinositol 3‐kinase, fibroblast growth factor receptor 1 and phosphatase and tensin homolog. However, there are also about 30%–40% of patients without driver mutations.^7^ Frequently, these patients fail in first‐line platinum salt and paclitaxel chemotherapy,^8^ and given their poor prognosis, there is an unmet need to develop improved treatment algorithms based on an individual patient requirement. In fact, a recent US‐based cross‐sectional study of >106,000 NSCLC patients revealed that less than 10% of LC patients are given targeted therapies. However, 85% of NSCLC patients are tested for common driver mutations.^9^

With the advent of enabling technologies, it is possible and cost effective to collect genomic, genetic and kinome profiling data and use this information for precision oncology that is tailored to an individual patient's demand. In fact, we are empowered to extend the therapeutic scope of kinase inhibitors (KI) beyond oncogenic driver mutations, and to expand the spectrum of personalized treatment schemes for inoperable LC patients by utilizing single or combined treatment algorithms. The same approach is used for patients who acquired drug resistance following specific KI use.

Undoubtedly, sustained signalling is one of the hallmarks of tumour growth,^10^ and deregulation of kinases provides a strong molecular rationale to broaden the perspective of kinase inhibitor–based therapies. So far, the possibilities to block signalling events in cancer patients have been underutilized, and with >80 Food and Drug Administration (FDA)–approved kinase inhibitors,^11^ alternatives exist to develop personalized therapies by targeting aberrant signalling pathways. Depending on the size and anatomical location, neoadjuvant therapies may also support the down‐staging of tumours, and therefore, patients become eligible for surgical interventions.

Here, we describe the prospect to develop new treatment algorithms based on 24 kinase targets, all of which were significantly upregulated in resectable lung tumours. So far, 83 FDA‐approved drugs are available which inhibit these 24 kinases. Yet, only 20 drugs are used for the treatment of NSCLC and are primarily inhibitors of EGFR, RET receptor (RET), ALK and MET.

We and others reported the utility of predictive and prognostic blood borne miRNA candidates for precision medicine in LC with predictive biomarkers allowing an evaluation of treatment response to adjuvant or neoadjuvant therapy.^12^, ^13^ Moreover, certain miRNAs inform on the histological subtype of LC.

Together, we investigated 95 LC patients diagnosed with AD and SQ, neuroendocrine tumours (NET) and metastatic tumours (MT), and performed whole‐genome transcript profiling to identify disease‐regulated genes and miRNAs. Through bioinformatics, we identified disease‐dependent gene–miRNA regulatory networks, and based on kinome profiling and signalling network analysis, we identified kinases, which function as master regulators (MRs) in such networks. Our study provides a strong rationale for their inhibition, and we propose new and personalized treatment algorithms of LC to broaden the perspective of precision oncology.

## 2 METHODS

### 2.1 Patients and specimens

The ethics committee of Hannover Medical School (MHH) approved the use of tissue resection materialof LC patients who received surgery at MHH during the period 2017–2022 (approval ID: 3381‐2016). We obtained informed consent from all patients. Board‐certified pathologists examined matched non‐tumours adjacent and tumour tissue, and we summarize the clinicopathological data in Table 1 and Table S1.

The study cohort of 95 LC patients consisted of 54 adenocarcinomas (22f, 32m); 23 SQ (9f, 14m); 9 neuroendocrine (4f, 5m) and 9 metastatic tumours (4f, 5m). In regards to the NET, there are four and two cases, respectively, of large‐cell and small‐cell carcinoma and three carcinoids. The lung metastatic tumours originate from three renal cell carcinoma, two colorectal cancers, one melanoma, one endometrial, one cholangiocarcinoma and one neuroendocrine cancer. Further information is given in Table S2.

### 2.2 RNA extraction

We used the miRNeasy Mini Kit (Qiagen) to isolate total RNA from frozen lung tissues according to manufacturer's recommendation and determined its quantity and purity with the NanoDrop ND‐1000 system. Additionally, we evaluated the purity of RNA with the nanoVette system (Beckman Coulter DU 730 UV/Vis Spectrophotometer) and considered RNA extracts with a ratio of ∼2.0 at 260/280 nm as acceptable for further analysis. Moreover, we assessed the integrity of the 18S and 28S ribosomal bands on denaturing agarose gels, and representative examples are shown in Figure S1.

### 2.3 Whole‐genome gene expression profiling

We used the GeneChip 3’IVT Plus reagent kit (Thermo Fischer Scientific) according to the manufacturer's recommendation and initiated first‐ and second‐strand cDNA synthesis with 100 ng of total RNA. We performed in vitro transcription with biotinylated ribonucleotide analogues and purified cRNA with magnetic beads. We quantified cRNA in a Beckman Coulter DU 730 UV/Vis Spectrophotometer (Beckman Coulter) and estimated the absorption ratio at 260/280 nm. We prepared cleaved cRNA with the fragmentation buffer and determined the size of the fragmented biotinylated cRNA by denaturing agarose electrophoresis. For this purpose, we mixed 9.4 µg labelled cRNA with 3′ fragmentation buffer and incubated the samples at 94°C for 35 min, followed by an incubation step at 4°C for ≥2 min. We assessed the quality of fragmented cRNA on denaturing agarose gels. Typically, we obtained fragments of the size of about 100 nt.

We hybridized 6 µg of the fragmented cRNA onto the GeneChip® HG‐U133 array strips and placed them into a GeneAtlas Hybridization Station according to manufacturer's instruction. Following hybridization at 45°C for 16 h, we initiated the washing and staining step in a GeneAtlas® Fluidics Station. Subsequently, we scanned the microarrays on a GeneAtlas® Imaging Station. We processed microarrays, which passed the quality controls (polyA, oligoB2 and 20XEHC) and performed in‐depth data analysis as described below.

### 2.4 Whole‐genome miRNA profiling

We labelled 700 ng of total RNA with the FlashTag^TM^ Biotin HSR RNA Labelling Kit (Thermo Fisher Scientific) according to the manufacturer's instructions. The assay involves a two‐step reaction, that is, poly(A) tailing and biotin ligation. The labelled samples were hybridized onto the Affymetrix GeneChip® miRNA array 4.1 (Affymetrix), at 48°C for 20 h. Subsequently, we placed the arrays into the GeneAtlas® Fluidics Station and initiated the wash and stain protocol according to the manufacturer's recommendation. We scanned the microarrays with the GeneAtlas® Imaging Station and processed the images with the GeneAtlas Instrument Control Software (Affymetrix) to generate CEL files. We processed microarrays, which passed the quality controls (polyA, oligoB2 and 20XEHC) and performed in‐depth data analysis as described below.

### 2.5 Lung cancer kinome profiling

The assay is based on the PamGene technology (www.pamgene.com), which enables the real‐time monitoring of kinase activity. Essentially, the assay determines the phosphorylation of canonical peptides of kinases. We performed kinome profiling for 54 patients by considering tumour and histologically proven adjacent non‐tumour tissue, and we compile information regarding patient demographics and tumour pathology in Table S2.

We performed all reactions on ice and placed frozen tissue of the size of about 1–3 mm^3^ into a Eppendorf tube. We added 100 µL M‐PER buffer (Thermo Fisher Scientific), which contained 2× protease and phosphatase inhibitor cocktails (Thermo Fischer Scientific) and gently mixed the samples. We incubated the lysates on ice for 60 min, followed by a centrifugation step at 4°C and 15 000 ×*g* for 15 min and collected 10 µL aliquots of the supernatant. These were immediately snap‐frozen and stored at −80°C. We determined the protein concentration with the Bradford assay (Thermo Fischer Scientific) and performed protein tyrosine (PTK) and serine/threonine kinase (STK) assays according to the manufacturer's protocols using the PamStation 12 platform (PamGene International BV) as previously described.^14^ For the PTK assays, we added 1x PK buffer, 1% BSA (bovine serum albumin), 1 M DDT (dichloro‐diphenyl‐trichloroethane) solution, 10% of PTK additive and 4 mM ATP (PTK reagent kit; article 32112.5, PamGene) to 5 µg of protein lysate in a final volume of 20 µL. The PTK assay buffer contained a fluorescein isothiocyanate (FITC)‐conjugated PY‐20 antibody, which recognizes phosphorylation sites of 196 peptides. For the STK assay, we added 1 µg of protein lysate 1x PK buffer, 1% BSA and 4 mM ATP (provided by PamGene, STK kit) to 1 µg of protein lysate in a final volume of 20 µL. The STK chip allowed an assessment of 144 peptides simultaneously. Unlike the PTK assay, we added a FITC‐conjugated antibody at the last cycle of the STK assay (Cycle 94) and measured the signal of the antibody, which recognizes phosphorylated peptides. We analysed the data with the Bio Navigator software version 6.2 (PamGene International BV).

### 2.6 Data processing

### 2.7 Identification of differentially expressed genes and miRNAs

#### 2.7.1 Hannover cohort

The genomic analysis consisted of 56 LC patients (Table S1), and included 32 AD, 14 SQ, 4 NET and 6 MT cases. Additionally, the genome‐wide miRNA profiling involved 70 cases. Here, the cohort consisted of 43 AD, 14 SQ, 6 NET and 7 MT cases. Table S2 provides an overview of the patient demographics and diagnostic information (pathology, clinical stage etc.). Most data sets are matched, that is, for the same biopsy, we obtained genomic and miRNA data.

We uploaded CEL files to the Transcriptome Analysis Console 4.0.2 (Thermo Fisher Scientific) and the geneXplain platform. We normalized the data with the Robust Multi‐array Average method and separately computed the principal component analysis for the gene and miRNA expression data (Figure S2). This defined 8% and 10% of cases, respectively, as outliers, that is, the samples were intermingled with controls (non‐cancerous adjacent tissue (NATs)). Consequently, outliers were excluded from further analysis. To find differentially expressed genes (DEGs) and miRNAs (DEMs), we computed the linear models for microarray data (LIMMA) package and corrected for multiple testing by applying a false discovery rate (FDR) at alpha 0.05. Only DEGs/DEMs with an FDR‐adjusted *p*‐value < 0.05 and a |fold change (FC)| ≥ 2 qualified for in‐depth analysis.

#### 2.7.2 The Cancer Genome Atlas Program (TCGA) cohort

We used the TCGA biolinks package to download raw counts for genes and miRNA expression data.^15^ Only matched data sets, that is, LC patients with gene and miRNA information were included. The lung adenocarcinoma (LUAD) data sets consisted of 524 cases and 58 non‐cancerous adjacent tissue (NATs). Similarly, the lung squamous carcinoma (LUSC) data sets consisted of 497 cases and 51 NATs.^15^ We used LIMMA^16^ to normalize the data and to define DEGs/DEMs by applying the following criteria: FDR‐adjusted *p*‐value < 0.05, a |FC| ≥ 2 in ≥50% of cases. All calculations were performed in R version 4.3.0.^17^ We separated the data by clinical stages and compared significant DEGs between the Hannover and TCGA cohorts. Of note, the DEGs are derived from two different platforms, that is, microarray and RNAseq, and although RNAseq revealed more DEGs in LC patients, >70% of DEGs are common between the two cohorts. We only considered commonly regulated genes of the two genomic platforms and selected top‐ranking ones for gene enrichment analysis, that is, KEGG (Kyoto Encyclopedia of Genes and Genomes) and hallmark gene sets.

### 2.8 Identification of differentially expressed kinases

We used the BioNavigator software version 6.2 (PamGene International BV) to normalize the data and to identify significantly regulated kinases. The BioNavigator software converts image files to signal intensities and corrects for local background noise. For the PTK assay, and for a given phosphopeptide, we fitted time‐resolved measurement cycles to estimate an initial phosphorylation rate (Figure S3), whereas for STK assay, the signals of the last cycle were quantified. We used LIMMA to identify significantly regulated kinases by comparing their activity in tumours versus NATs. We used the following criteria to define significantly regulated kinases: FDR‐adjusted *p*‐value < 0.05 and a |FC| ≥ 1.5.

### 2.9 MiRNA–gene regulatory networks

We used the miRNet 2.0 database to search for target genes of DEMs. The database contains comprehensive information on experimentally validated miRNA targets (https://www.mirnet.ca/miRNet/home.xhtml).^18^ We considered DEMs of lung tumours to search for putative gene targets and used the information as input files to search for database entries in miRNet 2.0. We compared the list of putative target genes with DEGs of LC patients as determined in our genomic analysis, and this allowed us to identify validated DEG targets and to construct miRNA–gene regulatory networks. Subsequently, we visualized the networks with the software Cytoscape 3.9.1.^19^ Additionally, we computed correlations between DEMs and DEGs. Based on the normality test, we calculated Pearson and/or Spearman correlations. We compared the results to the miRNet proposed targets. Furthermore, we performed multiple query types in miRNet by searching for potential miRNA targets based on patient‐specific DEGs (transcriptomic analysis), and vice versa, used patient‐specific miRNAs as input file to search for potential DEG targets.

### 2.10 Survival analysis

For the survival analysis, we included 497 LUAD and 496 LUSC patients with OS information (https://xenabrowser.net/). The start of the survival analysis was defined as a definitive diagnosis of LC, the earliest years were 1991 (LUAD) and 1992 (LUSC), and the survival analysis ended in 2015 (LUAD) and 2013 (LUSC). The median follow‐up time was 21.9 (LUAD) and 21.8 (LUSC) months, and the Interquartile range (IQR) was 23.5 (LUAD) and 31.4 (LUSC) months. We divided the patients into high‐ and low‐expression individuals according to the median value of the gene/miRNA expression, and constructed Kaplan–Meier curves to determine overall survival (OS). We performed log‐rank test and univariate Cox proportional hazards regression analysis to determine statistical significance and hazard ratio (HR) estimate with 95% confidence interval (CI). To evaluate the proportional hazard assumption, we computed the Schoenfeld residuals in R (Supporting Information 1) and assessed linearity of the Cox proportional hazard regression analysis by calculating fractional polynomials.

### 2.11 Search for master regulatory kinases in canonical pathways of LC patients

We uploaded DEGs with a |FC| ≥ 3 onto the geneXplain platform, and applied the function ‘find master regulators’. The underlying genetic algorithm has been published^20^, ^21^ and MRs were identified based on TRANSPATH® database entries. We only considered MRs with an FDR‐adjusted *p*‐value < 0.05 and used default settings of the algorithm, that is, a score of >0.2. The score defines the connectivity of the MR with other molecules of the same pathway, and we used a threshold >1 for the *Z*‐score which measures specificity of the MR. We adjusted the algorithm to a maximum radius of 10 molecules upstream of input DEGs, and the software identified MRs in different canonical pathways. Next, we searched for significantly regulated kinases which qualify as MRs and identified pathways specific to a histological type of LC (AD, SQ, NET and MT). The kinase/kinome activity data of lung tumour samples informed on the possibilities to block signalling pathways by targeting hyperactive kinases. Finally, we constructed regulatory signalling networks, which consisted of DEGs, DEMs targeting the DEGs and kinases.

### 2.12 Statistics

We used R and the geneXplain (https://genexplain.com/) software to perform statistical analysis. If not otherwise specified, all tests were two‐tailed, and an FDR‐adjusted *p*‐value < 0.05 was considered to be statistically significant.

## 3 RESULTS

**FIGURE 1:**
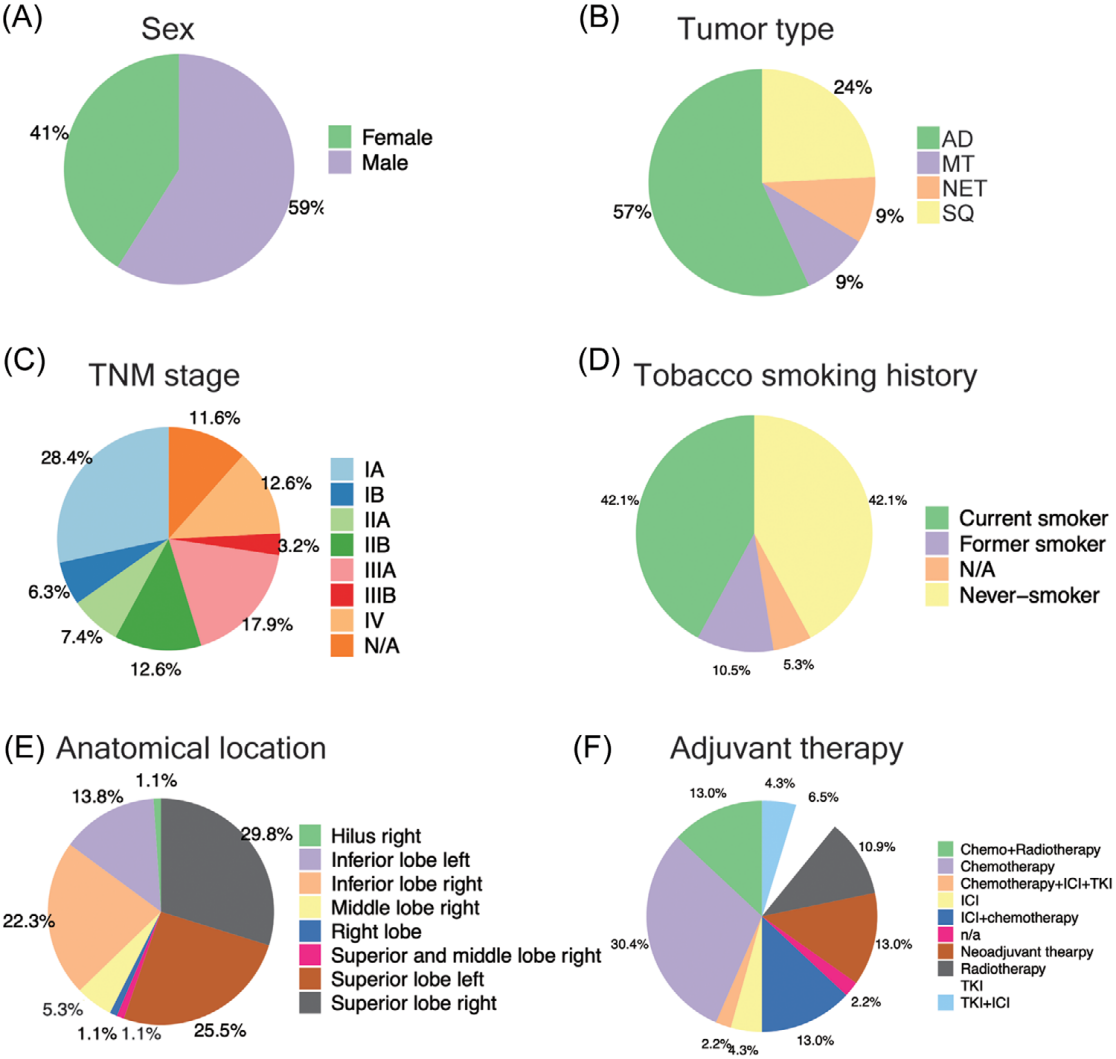
Patient demographics. (A) Gender of patients. (B) Distribution of the histological types of lung cancer. (C) Distribution of TNM stages. (D) Tobacco smoking history. (E) Anatomical location of the tumour. (F) Adjuvant therapeutic interventions.

Our investigation conforms to a three‐arm study design and consisted of a genomic, miRNA and kinome profiling arm. We summarize the patient demographics in Figure 1, and this includes sex, tumour type, TNM stage, tobacco smoking history, anatomical location and drug therapies. The majority are AD cases and the cohort consisted of slightly more males (59%) than females (41%). Furthermore, 72.6% patients are TNM Stages I–IIIA and based on self‐reporting, the tobacco smoking history is surprisingly similar, that is, 42.1% and 42.1% of current and never smokers. In regards to tumour location, there are three major anatomical regions: inferior and superior right and superior left lobe. Majorly, the patients received adjuvant drug therapy (mainly clinical stages ≥2) which consisted of chemo, radio and targeted therapies. About 20% of patients were treated with ICIs either as standalone or a combination of chemo and targeted therapy, and 13% of patients received neoadjuvant therapy primarily consisting of chemo‐ and radiotherapy. See Tables S1 and S2 for further information.

### 3.1 Genomic profiling of different LC types

To identify disease‐regulated genes (DEGs) and miRNAs, we compared the genomes of tumours to NATs. Shown in Figure S2 is the principal component analysis (Panel A), and the heatmaps (Panel B) clearly segregated tumours from NATs across the different histological LC subtypes. We identified 439, 1240, 383 and 320 significantly upregulated genes for AD, SQ, NET and MT cases, respectively, and there are 1092, 1477, 609 and 1267 downregulated DEGs (Table S3). Common to all tumours is the predominant repression of gene expression.

**FIGURE 2:**
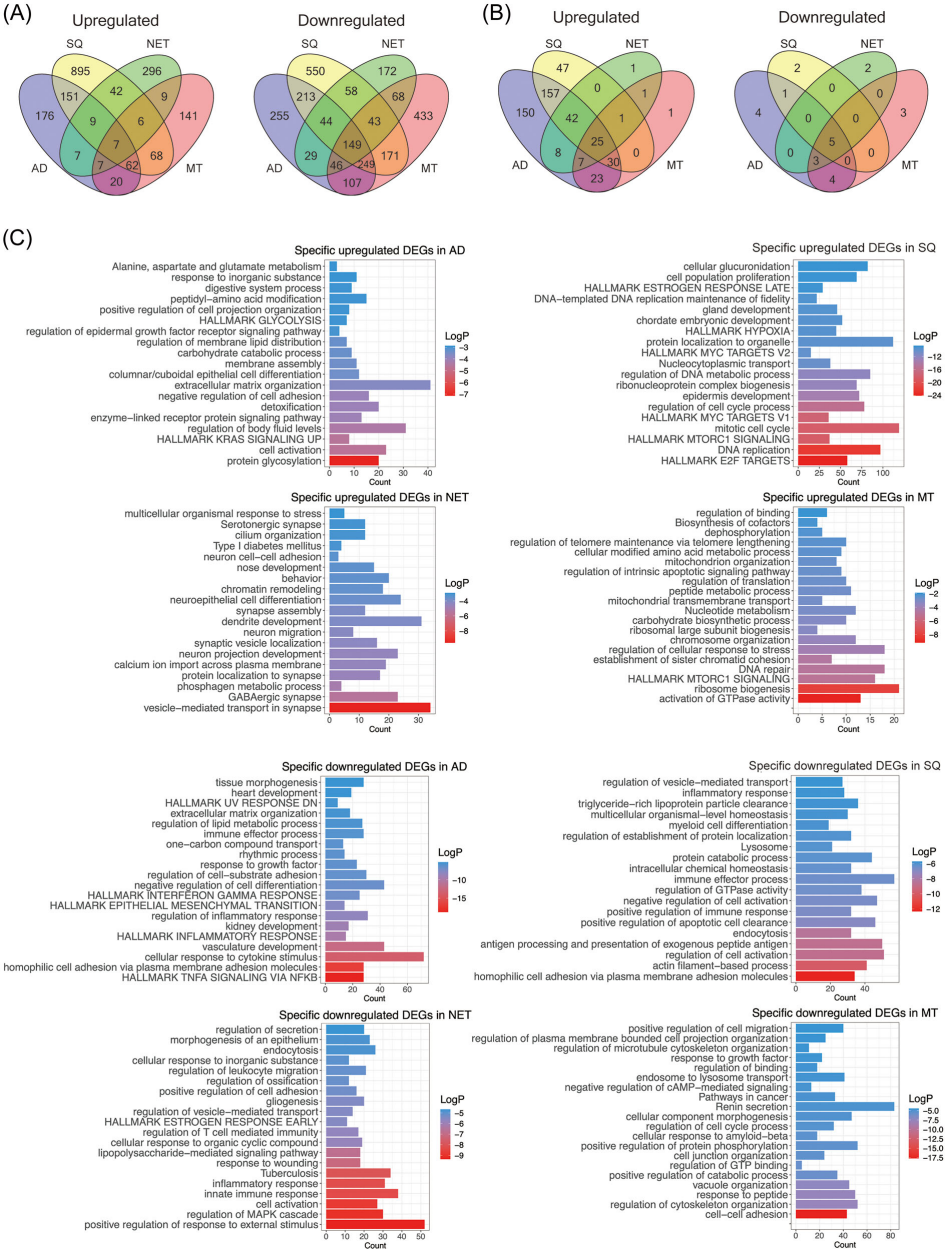
Genome profiling of the various types of lung cancer. (A) Venn diagrams of up‐ and downregulated genes in AD, SQ, NET and MT tumours. (B) Venn diagrams of up‐ and downregulated miRNAs in AD, SQ, NET and MT tumours. (C) Bar plots showing significantly enriched gene ontology terms and hallmark gene sets of DEGs specifically up‐ and down regulated in AD, SQ, NET and MT tumours. AD, adenocarcinoma; MT, metastatic tumours; NET, neuroendocrine tumours; SQ, squamous cell carcinoma.

To identify DEGs specifically asscociated with a histological subtype of LC, we compared the genomes of AD, SQ, NET and MT cases to NATs. This defined 176, 895, 296 and 141 genes specifically upregulated and 255, 550, 172 and 433 repressed ones in the different LC subtypes as shown in Figure 2A.

Furthermore, we identified 442, 302, 85 and 88 significantly upregulated miRNAs for AD, SQ, NET and MT cases, and for the same patients, there are 17, 8, 10 and 15 downregulated miRNAs. In addition, we identified 150, 47, 1 and 1 specifically upregulated and 4, 2, 2 and 3 downregulated miRNAs in AD, SQ, NET and MT (Figure 2B). Collectively, miRNAs are primarily upregulated in tumours, whereas transcriptomes are predominantly repressed (Figure 2B and Table S4).

We used Metascape as an annotation tool for DEGs which were specifically regulated in the various histological subtypes of LC, and we compared significantly enriched terms between them. Note, in this comparison we used non‐overlapping genes across histological subtypes. As shown in Figure S4, and with the exception of mTORC1 signalling, which is common to SQ and MT, none of the terms overlapped. Therefore, the gene ontologies and enriched terms are specific for a given histological subtype of LC.

For AD, and among unique terms associated with upregulated DEGs, we wish to emphasize KRAS and EGFR signalling and protein glycosylation. Significantly enriched terms for SQ are E2F and MYC targets, hypoxia and oestrogen response and for NET tumours, neuroepithelial cell differentiation and GABAergic signalling. Finally, enriched terms for MT are MTORC1 signalling, activation of GTPase activity, chromosome organization and DNA repair. We also considered enriched terms for downregulated DEGs. Here, TNFα signalling, response to cytokine stimulus, actin filament‐based process and immune response, regulation of MAPK cascades and cell adhesion were specific for AD, SQ, NET and MT tumours (Figure 2C).

**FIGURE 3:**
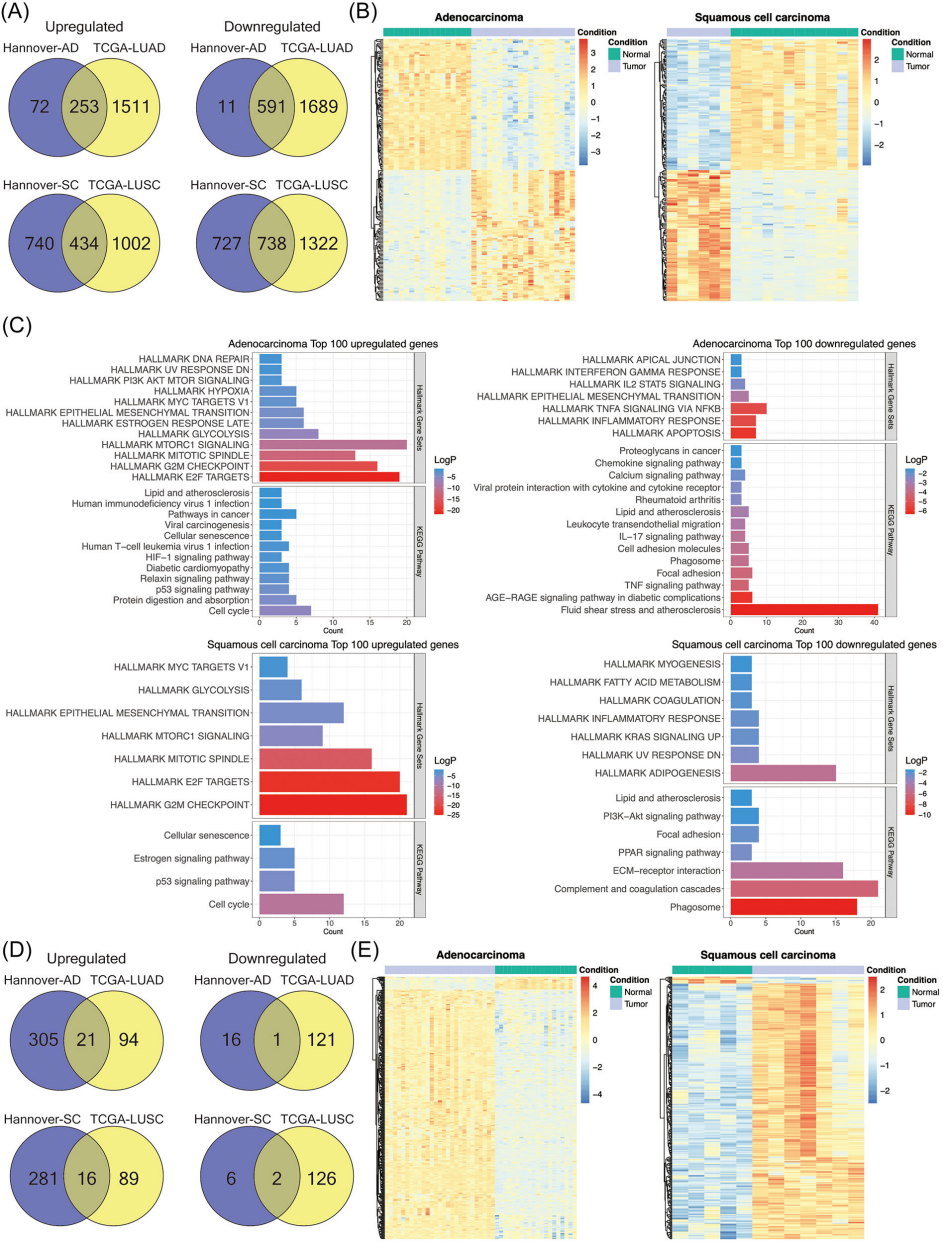
Independent validation of lung cancer genomics. (A) Venn diagrams of commonly regulated genes in AD and SQ between the Hannover cohort (test set) and TCGA cohort (validation set). (B) Heatmaps of DEGs of the Hannover cohort. The DEGs of the non‐tumorous adjacent tissue (NATs) are clearly separated from the tumour‐associated DEGs, and the colour codes represent *Z*‐scores. (C) Bar plots of the significantly enriched hallmark gene sets and KEGG pathways for the top 100 DEGs in adenocarcinoma and squamous cell carcinoma of the Hannover cohort. (D) Venn diagrams of the commonly regulated miRNAs in AD and SQ between the Hannover (test) and TCGA (validation) cohorts. (E) Heatmaps of the DEMs of the Hannover cohort. The DEMs of the NATs are clearly separated from the tumour associated DEMs, and the colour codes represent *Z*‐scores. DEGs, differentially expressed genes; DEMs, differentially expressed miRNAs.

To independently validate the results of the present study (“Hannover cohort”), we retrieved data from the TCGA database (https://www.cancer.gov/ccg/research/genome‐sequencing/tcga) and applied the following criteria: DEGs were defined with an FDR‐adjusted *p*‐value < 0.05, a |FC| ≥ 2 in ≥50% of cases. Note, the TCGA source does only compile LUAD and LUSC cases. Therefore, we compared 524 LUAD cases to 58 NATs and separated the data by clinical stages. We searched for common DEGs between the two cohorts and found > 70% of the up‐ and downregulated DEGs as commonly regulated. The results are similar when compared to the different histological subtypes of LC (Venn diagram, Figure 3A and Table S5). Therefore, the data agreed reasonably well.

Depicted in Figure 3B are the heatmaps for DEGs of the Hannover cohort, and the colour codes represent *Z*‐scores which measures the standard deviations from the mean of a gene expression values. We obtained perfect segregation of AD and SQ tumours from NATs.

Subsequently, we searched for enriched gene ontologies with the Metascape software^22^ and selected the top 100 regulated genes common between the Hannover and TCGA cohorts. The FC of the selected genes ranged from 3‐ to 14‐fold. Additionally, we used hallmark gene sets and KEGG pathways and show the results in Figure 3C. For upregulated genes, the hallmark gene sets underscored mTOR signalling, G2M checkpoint, E2F targets and glycolysis in LUADs (AD cases). Similarly, KEGG pathway analysis emphasized cell cycle, p53 signalling, cellular senescence and cancer pathway.

In regards to downregulated genes (range 5–20‐fold), the hallmark gene sets highlighted apoptosis, inflammatory response and TNFα signalling via NFKB. We obtained similar results for KEGG pathways, and prominent examples of enriched terms were TNFα signalling, immune and chemokine signalling and leukocyte migration (Figure 3C).

In the same way, we analysed SQ tumours (LUSC), and for upregulated DEGs (range 6–194‐fold), the hallmark gene sets were similar to AD cases, that is, mitotic spindle, E2F targets, G2M checkpoints and mTOR signalling, while KEGG pathway analysis highlighted p53 signalling, cell cycle and cellular senescence. For repressed DEGs (range 9–72‐fold), the hallmark gene sets emphasized inflammatory response, KRAS signalling and DNA repair, whereas KEGG highlighted phagosome, complement and coagulation cascades and ECM receptor interactions. We summarize the results in Table S5.

Unlike the cancer transcriptomic data described above, there was little overlap among DEMs of the Hannover and TCGA cohort (Figure 3D). In fact, for AD only 6% of up‐ and downregulated DEMs are common to both cohorts, whereas for SQ 5% and 25% DEMs are commonly regulated.

### 3.2 Gene networks in different histological subtypes of LC

**FIGURE 4:**
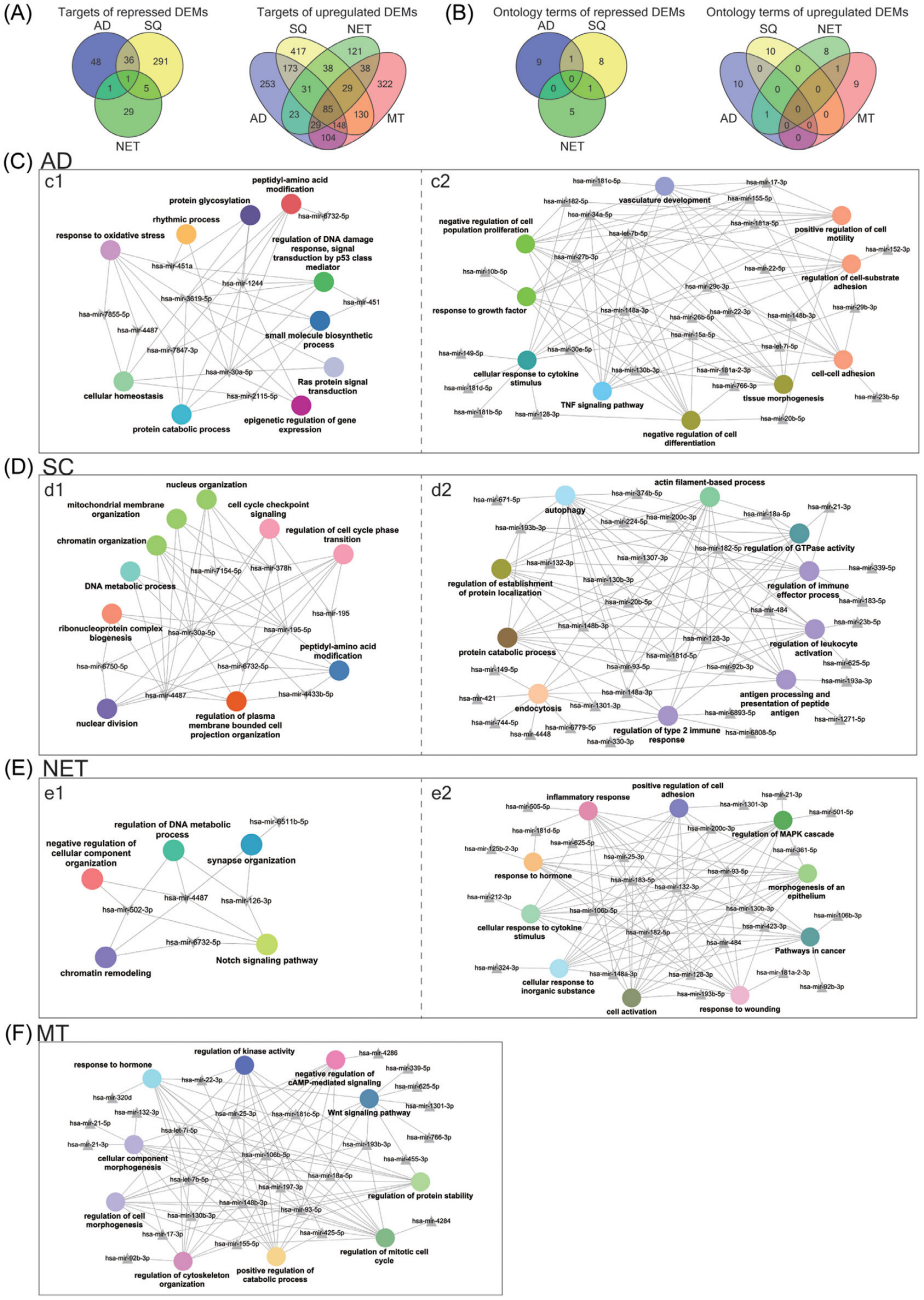
MiRNA–gene networks in various histological types of lung cancer (LC). (A) Venn diagrams showing the number of differentially expressed genes (DEG) across different histological types of LC which are the targets of up‐ and down regulated differentially expressed miRNAs (DEMs). (B) Venn diagrams of significantly enriched gene ontology across different histological types of LC which are the targets of up‐ and down regulated DEMs. (C) Regulatory miRNA‐gene networks of lung adenocarcinomas based on Metascape analysis of upregulated DEGs which are the targets for repressed DEMs (c1) and downregulated DEGs which are the targets for upregulated DEMs (c2). (D) Regulatory miRNA–gene networks of lung squamous carcinoma based on Metascape analysis of upregulated DEGs which are the targets for repressed DEMs (d1) and downregulated DEGs which are the targets of upregulated DEMs (d2). (E) Regulatory miRNA–gene networks of lung neuroendocrine tumours based on Metascape analysis of upregulated DEGs which are the targets for repressed DEMs (e1) and downregulated DEGs which are the targets of upregulated DEMs (e2). (F) Regulatory miRNA–gene networks of metastatic lung tumours based on Metascape analysis of upregulated DEGs which are the targets for repressed DEMs (f).

To gain insight into the molecular wiring across different histological subtypes of LC, we constructed regulatory gene networks, and to ascertain the relationship between DEMs and DEGs, we analysed data deposited in the miRNet database. This revealed experimentally proven miRNA gene targets which we compared to DEGs specific for the different subtypes of LC (Figure 2A). Based on the paradigm that miRNAs repress the expression of gene targets, we identified 253, 417, 121 and 332 DEGs specific for AD, SQ, NET and MT (Figure 4A). Note, all the target genes were repressed in lung tumour samples (Figure 2A). Conversely, for repressed DEMs, we discovered 48, 291, 29 and 0 upregulated DEGs. Furthermore, there are 85 targets in common and therefore were irrespective of the histological subtype of LC. The gene ontologies emphasized UV response (DNA repair), hallmark gene set EMT and TNFα signalling for upregulated DEMs (Figure 4A). For repressed DEMs, only one target gene is common, that is, nucleoside diphosphate kinase 1 which we found upregulated in all lung tumour samples, and this kinase supports metastatic spread.

Additionally, we computed correlations between DEMs and DEGs. Based on the normality test, we calculated the Pearson and/or Spearman correlations and summarize the results in Table S6. In regards to AD, we observed 1231 significant correlations. We compared the results to the miRNet proposed targets and found 846 DEGs (64.2%) to be in common. For SQ, we computed 1425 significant correlations, and when compared to the miRNet targets there are 947 DEGs (50.9%) in common. Together, the correlation analysis identified more potential miRNA‐DEG associations.

Furthermore, we computed multiple query types by searching for potential miRNAs which target patient specific DEGs (Hannover cohort data). Additionally, we used significantly regulated miRNAs of the Hannover cohort data as input file to search for potential DEG targets. We compared the results, and for ADs 96% of the targets are in common based on the multiple query approach, and similar results were obtained for SQ, that is, 98% (Figure S5A). We used Metascape gene annotations to construct regulatory miRNA–gene networks. We selected the top 10 biological processes based on FDR‐adjusted *p*‐values and required the term to cover > 80% of DEGs specific for a given tumour subtype (Figure 4A). We likewise selected the top 10 miRNAs based on the number of target genes for each biological process and compiled the individual data in Table S6. In this way, we constructed miRNA–gene networks specific for AD, SQ, NET and MT LCs. We compared the terms among the different types of LC, and the Venn diagram in Figure 4B shows the non‐overlapped terms among the various histological subtypes of LC. We obtained similar results for repressed DEMs. Here, we considered terms associated with upregulated DEGs. In fact, in the comparison of AD versus SQ, there is only one term in common, that is, peptidyl amino acid modification. Together, this demonstrates specificity for the constructed miRNA–gene networks.

Shown in Figure 4C–F are the regulatory miRNA–gene networks for the different histological types of LC, and we used Cytoscape to create the network. Although the terms are generic in nature, the underlying DEGs are highly specific and encompass potential drug targets. For instance, for the SQ network (Figure 4D1), the Metascape analysis revealed cell cycle phase transition as a statistically significant enriched term and embedded in this term are highly regulated DEGs, notably CDK1, CHEK1 and GSK3B which were induced by eight‐, four‐ and twofold, respectively, in expression. Strikingly, the activities of the coded kinases were also significantly increased in the same patient derived tumour biopsies (see below). A further example relates to peptidyl‐amino acid modification, and among genes embedded in this term is the fivefold upregulated NTRK2. Importantly, the kinase activity of NTRK2 is also significantly increased, and its inhibitors entrectinib and larotrecitinib are already approved for the treatment of solid tumours in patients diagnosed with NTRK2 fusion proteins.

Together, the miRNA–gene networks bear great relevance for an identification of potential drug targets.

#### 3.2.1 Kinome profiling in LC

**FIGURE 5:**
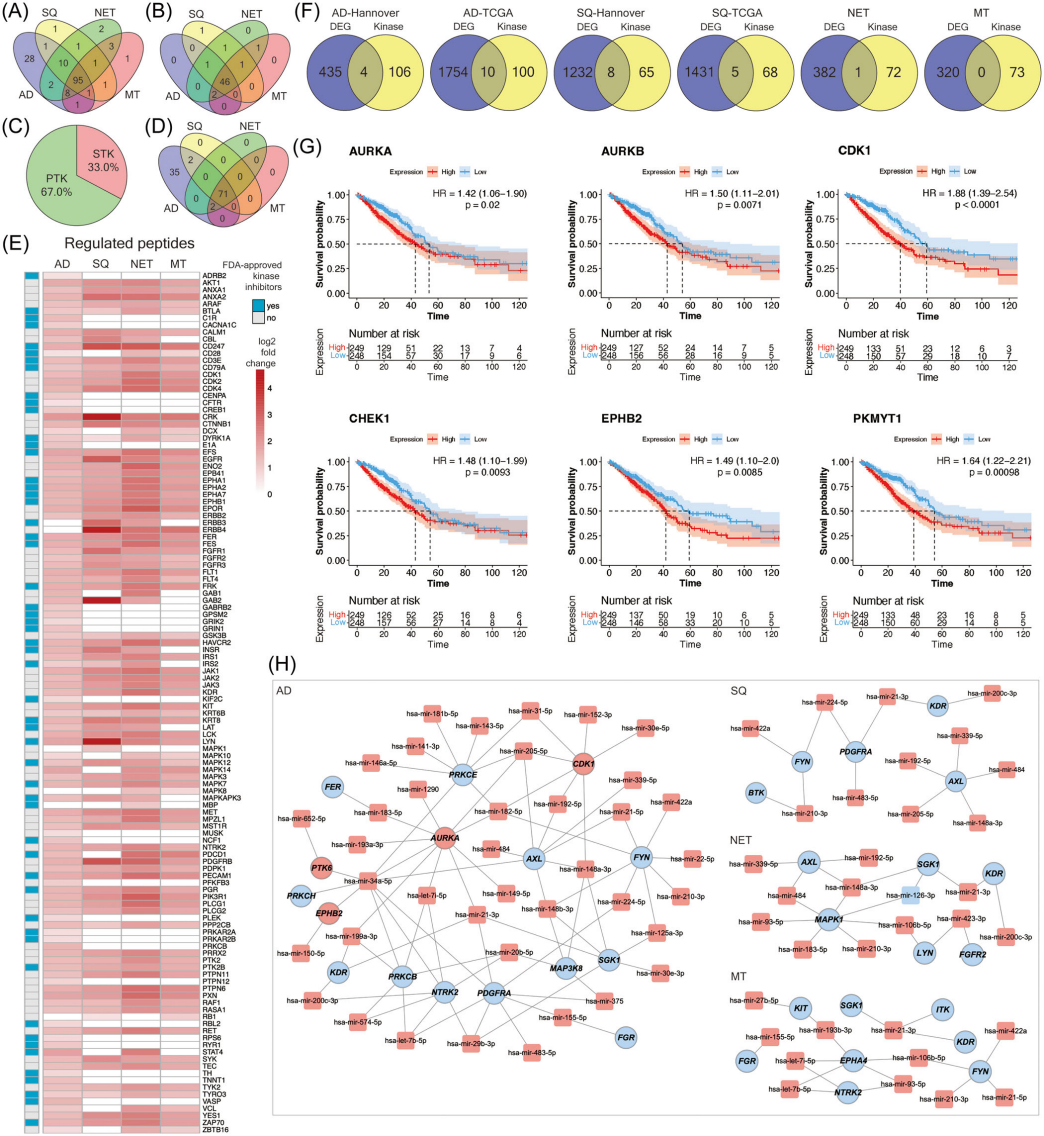
Kinome profiling of lung cancer. (A) Venn diagram showing the distribution of phosphorylated peptides across adeno, squamous, neuroendocrine and metastatic lung tumours. (B) Venn diagram of significantly regulated kinases whose activity was directly measured. (C) Pie chart of commonly regulated PTKs and STKs between the various histological types of lung cancer. (D) Venn diagram of regulated kinases based on upstream analysis across the various histological types of lung cancer. (E) Kinase activities across different histological types of LC. The data are log2 fold changes, and the blue quadrants show FDA‐approved kinase inhibitors for a given kinase. (F) Venn diagrams of DEGs coding for regulated kinases across the various types of LC. (G) Kaplan–Meier survival plots of DEGs coding for significantly regulated kinases. The kinase activities were determined in the Hannover cohort, and the DEGs stem from the Hannover test set and the TCGA‐LUAD validation set. (H) Regulatory miRNA‐gene networks for DEGs coding for significantly regulated kinases. Red colour: upregulation. Blue colour: downregulation. DEGs, differentially expressed genes; FDA, Food and Drug Administration; LC, lung cancer; PTKs, protein tyrosine kinases; STKs, serine/threonine kinases.

We used the PamGene® platform to identify aberrant kinase activities in tumours and NATs of 54 patients. The assay measures the phosphorylation of peptides, and each peptide contains canonical amino acid sequences which are recognized by distinct serine/threonine and tyrosine kinases. We obtained two sets of data. First, direct measurement of the phosphorylation of kinases, and second, an identification of regulated kinase based on the phosphorylation of canonical amino acid sequences. Here, the upstream kinase phosphorylates a specific substrate, and therefore, the phosphopeptide is a read‐out of the kinase activity. Depicted in Figure 5A is a Venn diagram of phosphorylated peptides across the different histological types of LC. We measured an increased phosphorylation of 156 peptides with 95 being common between the different types of LC (Figure 5A). Conversely, there are 28, 1, 2 and 1 phosphorylated peptides uniquely associated with AD, SQ, NET and MT tumours. Of the 156 phosphorylated peptides, there are 72 which allow direct measurement of regulated kinases. Note, for some kinases, such as the EGFR, we measured the phosphorylation of several peptides which contain specific tyrosine residues, and in Figure 5B we show significantly regulated kinases between the different types of LC. Together, we identified 53 significantly regulated kinases of which 46 are common (Figure 5B and Table S7). We did not observe repressed kinase activities but upregulation of primarily PTKs, that is, 67% (Figure 5C), and identified 35 serine/threonine kinases specifically regulated in AD (Figure 5D), and these kinases phosphorylate 28 peptides (Figure 5A). Moreover, upstream kinase analysis revealed 71 kinases regulated in common (Figures 5D and S6a–d) of which 67.6% are tyrosine kinases, and in Figure S4 we show examples of kinetic plots for tumour and NAT biopsies. Converging the results of Figure 5B (direct measurement of 53 phosphorylated kinases) and upstream kinase analysis of phosphorylated peptides (see Figure 5D) yielded a total of 137 distinct kinases which were significantly upregulated in LC (Figure S6e).

The heatmap shown in Figure 5E informs on tumour regulated kinases, and the data are log2 fold increases across different histological subtypes of LC. In Table S7, we specify the amino acid sequence of the target protein which becomes phosphorylated, and a more detailed information is given in Supporting Information 2.

Next, we addressed the questions whether the genes coding for the kinases were also regulated at the gene expression level and show the results in Figure 5F. There are 4 (PTK6, CDK1, AURKA and EPHB2) and 10 kinases (PAK1, PKMYT1, AURKB, CHEK1, CDK1, JAK3, EPHB2, PRKCG, PTK6 and AURKA) of the Hannover‐AD and TCGA cohort whose gene expression and kinase activities were upregulated in the same way. Similarly, there are eight (CDK1, CHEK1, EPHA4, GSK3B, MAP2K6, NTRK2, PAK1 and PTK2) and five kinases (CDK1, CHEK1, EPHB2, PAK1 and PKMYT1) in common between the two cohorts (Figure 5F). For neuroendocrine LC tumours, RET is the only kinase where the gene expression (11‐fold) and kinase activity (5‐fold) were increased in the same way. Together, the data imply that only a few kinases are simultaneously regulated at the gene expression and kinase activity level. Therefore, kinase profiling is of critical importance.

Additionally, we determined the prognostic value and computed univariate Cox regression proportional hazard models for genes that code for 110 significantly regulated kinases based on upstream analysis (Figure 5D and Table S9). We identified 37 kinase coding genes in AD with a statistically significant HR. However, only 18 kinase coding genes had a HR > 1 (range 1.1–1.79). For SQ, there are three kinase coding genes with a statistically increased HR (range 1.2–1.37) (Table S9). Together, the prognostic value of the univariate Cox regression proportional hazard models based on the expression of the kinase coding genes is less obvious.

Notwithstanding, we were able to compute meaningful Kaplan–Meier survival curves for kinases where the change in gene expression and kinase activity agreed, and depicted in Figure 5G are the plots for the kinases AURKA, AURKB, CDK1, CHEK1, EPHB2 and PKMYT1. Here, the prognostic value for these kinases could be established, and in the case of CDK1, clinical Phase II trials in NSCLC and SCLC are ongoing (NCT05651269 and NCT02161419).

Furthermore, for the different histological subtypes of LC, we constructed networks that describe the relationship between miRNAs, their gene targets and the activity of the kinase protein (Figure 5H). As an example, the expression of the genes coding for PDGFRA, KDR and NTRK2 kinase are downregulated, and the miRNAs targeting these kinases are upregulated. Although this fits the paradigm, that is, miRNAs repress gene translation, we assayed increased kinase activity, and this implies independent mechanisms for their regulation. We also identified upregulated miRNAs linked to an increased expression of kinase coding genes, as denoted for CDK1, ARUKA and PTK6, and their kinase activity was likewise induced. The results reinforce the notion that expression of the kinase coding gene and the activity of the coded protein cannot be correlated easily. However, the networks are of critical importance to define therapeutic targets as described below (see master regulatory networks).

Apart from miRNet searches, and given that the miRNA–DEG correlation analysis identified more potential targets (see above and Table S6), we searched for new kinases based on the correlation analysis. By applying a threshold of *R* ^2^ ≥ 0.7, we used the results of the correlation analysis to identify DEGs coding for kinases and compared the results with their actual enzyme activity, as determined by kinome profiling of tumour samples from LC patients. As shown in Figure S5B, none of the correlation analysis revealed additional DEGs coding for kinases whose enzyme activity was likewise upregulated in tumour samples of AD patients. However, for SQ patients, we identified six kinases of which five were also flagged as potential targets based on the miRNet query (Figure 5H). Together, the DEM–DEG correlation analysis identified the FGR kinase as an additional potential drug target for SQ (Figure S5b).

#### 3.2.2 Commonly regulated kinases in LC

**FIGURE 6:**
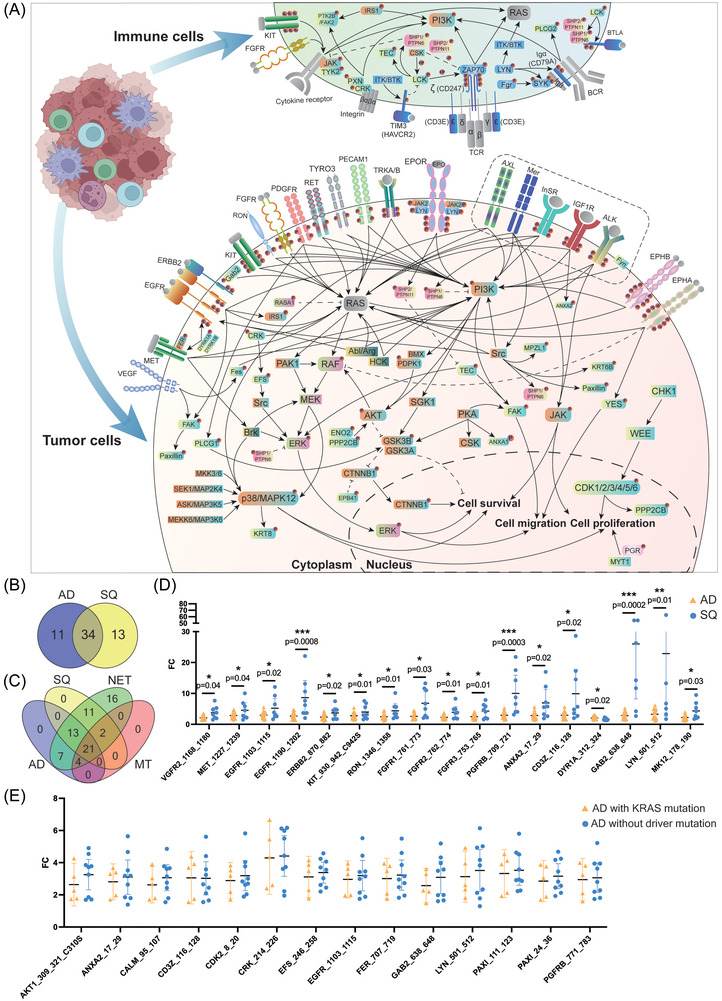
Identification of signalling networks in lung cancer. (A) Depicted are the signalling networks based on 92 commonly regulated kinases across different histological types of lung cancer. Kinases marked with ℗ represent direct phosphorylation of the protein. Rectangular shaped kinases are based on upstream kinase analysis. Grey coloured kinases were not measured. (B) Dot plots of significantly regulated kinases between lung adenocarcinoma and squamous cell carcinoma. The data are fold changes, and statistical testing is based on a two‐sided *t* test when the data are normally distributed; otherwise, we used the two‐sided Mann–Whitney *U* test. Error bars represent 95% confidence intervals. (C) Dot plots of significantly regulated kinases in lung adenocarcinoma patients with KRAS or without KRAS mutation. The data are fold changes, and statistical testing is based on a two‐sided *t* test when the data are normally distributed; otherwise, we used the two‐sided Mann–Whitney *U* test. Error bars represent 95% confidence intervals.

Depicted in Figure 6A are the complex signalling networks in LC, and we separate the signalling networks in tumour and immune cells. Based on the paradigm that ≥70% of patients show the same change, that is, induced kinase activity, we identified 45 and 47 significantly regulated kinases in AD and squamous LC cases of which 34 are regulated in common (Figure 6B, Venn diagram). We did not observe repressed kinase activities. In the same way, we analysed the neuroendocrine and metastatic tumours and identified 74 and 27 kinases as significantly upregulated. Additionally and irrespective of the histologic subtype of LC, we identified 17 tyrosine and 4 serine‐threonine kinases as commonly upregulated across 54 LC patients (Figure 6C).

As shown in Figure 6A, RAS is not regulated in the present study (grey coloured), while the rectangular marked proteins are based on upstream kinase analysis. Proteins marked by the symbol ℗ are direct measurements of phosphorylated kinases. Additionally, we identified regulated kinases by upstream analysis. Here, the phosphorylation of canonical peptides was assayed. An important finding of our study is an upregulation of a wide range of receptor tyrosine kinases in tumours such as the vascular endothelial growth factor receptor 2 (VEGF2/KDR), EGFR and ERBB2, ephrin type‐A&B receptor (EPHA & EPHB2), platelet‐derived growth factor receptor, the RET and the MER, MET and RON proto‐oncogenes. Additionally, we assayed peptides typically phosphorylated by the AXL (Tyro3‐Axl‐Mer (TAM) receptor) tyrosine kinase, the insulin (InSR) and insulin‐like growth factor 1 receptor (IGF1R), the ALK, neurotrophic TK, the kit proto‐oncogene and the fibroblast growth factor, and we obtained clear evidence for their increased activity in tumours of LC patients (range in AD 2–4‐fold, in SQ 2–24‐fold). Moreover, for some of the receptor tyrosine kinases, we determined the phosphorylation sites and show the downstream signalling of kinases which were regulated as well. For instance, we assayed the phosphorylation of three peptides of the EGFR: the amino acid sequence (AS) 1103–1115 (GSVQNPYHNQPL), the AS 1165–1177 (ISLDNPDYQQDFF) and AS 1190–1202 (STAENAEYLRVAP) and observed differences between the various histological subtypes of LC. The differences in the tyrosine phosphorylation can be explained due to variant SRC and ABL1 kinase activity with implications for downstream signalling events. Similarly, we assayed the tyrosine phosphorylation of the MAPK1 peptide HTGFLTEYVATRW (AS 180–192) which was significantly increased in SQ cases only (range 1.2–3.7‐fold). This peptide can be phosphorylated by the kinases RAF1, RET, JAK2, ERK1 and LYN, and all of them are upregulated in the Hannover cohort of LC patients. Likewise, we observed increased phosphorylation of the tyrosine residue 185 of the p38gamma/MAPK12 peptide ADSEMTGYVVTRW, and one study reported the precisely ordered phosphorylation reactions of the p38 mitogen‐activated protein (MAP) kinase which involves the kinases MKK3/6, MEKK6, SEK1 and ASK.^23^

#### 3.2.3 Tumour cell infiltrating lymphocytes

A major finding of our study is the unique regulation of kinases among tumour infiltrating lymphocytes. Importantly, these kinases were regulated in all tumours irrespective of the histological subtype. In regards to the T‐cell receptor (TCR), the data were complex. In general, the TCR αß and γδ heterodimers engage with CD3 molecules,^24^ and the tyrosine residues of the CD247/CD3ζ chain are of critical importance in activating TCR. We observed 10‐fold induced phosphorylation of the immunoreceptor tyrosine‐based activation motif (ITAM) KDKMAEAYSEIGM of CD247/CD3ζ. The phosphorylation of this ITAM is catalysed by the ZAP70 kinase, and we found its activity increased by three‐ to fourfold in LC. Moreover, the lymphocyte cell‐specific protein tyrosine kinase LCK phosphorylates the ZAP70 protein (ζ chain of TCR‐associated protein kinase 70), and we measured a fourfold increased LCK activity in tumour biopsies of LC patients. Additionally, the CD3ε polypeptide PVPNPDYEPIRKG is phosphorylated by LCK, and phosphorylation of this peptide was increased by fourfold. Together, we observed increased phosphorylation of the CD3ε and CD3ζ chains and measured increased activity of kinases catalysing the phosphorylation of these peptide chains. Obviously, this will support assembly of the TCR–CD3 complex.

The signalling roles of CD3ε were recently discovered and through a series of mechanistic studies, CSK and the protein tyrosine phosphatases SHP1 and SHP2 were shown to inhibit TCR signalling.^25^ We assayed the CD3ε‐specific peptides PVPNPDYEPIRKG at AS 182–194 and KGQRDLYSGLNQR at AS 193–205 and determined only for the first ITAM (AS 182–194) a fourfold increased phosphorylation. Furthermore, the phosphatases SHP1 and SHP2 are activated by tyrosine phosphorylation.^26^ We assayed the phosphorylation of the SHP1 at AS 558–570 KHKEDVYENLHTK and for SHP2 at AS 580–590 SARVYENVGLM, and for both peptides, we observed up to fourfold increased tyrosine phosphorylation. Moreover, the SHP1 peptide is phosphorylated by LCK, and its activity was upregulated by fourfold (Table S7). Furthermore, SHP2 regulates SRC family kinase activity and RAS/ERK activation by controlling CSK recruitment.^27^

Concerning the B‐cell receptor, the only peptide available on the PamGene platform is the ITAM of the Igα chain. Its tyrosine‐specific phosphorylation at AS 181–193 EYEDENLYEGLNL is catalysed by SYK, and this kinase is activated by LYN. We determined a four‐ and fivefold increased activities of the LYN and SYK kinases and observed an up to fourfold increased ITAM phosphorylation of the Igα chain in neuroendocrine LC cases. The data imply activation of the pro to the pre‐B‐cell stage but is confounded by the limited information available. Furthermore, BCR induces phosphorylation of PLC‐γ2 on tyrosine Y1217 which contributes to an activation of this phospholipase.^28^

A further example relates to the TEC tyrosine kinase which exerts multiple functions on the immune system and T‐cell signalling.^29^ We assayed the peptide RYFLDDQYTSSSG at AS 512–524 and found its activity increased by fourfold.

Collectively, while some essential components of the TCR complex were activated, inhibitors of TCR signalling were likewise upregulated, and the results are suggestive for a dysfunctional TCR. Interestingly, none of the gene coding for the TCR complex was significantly regulated in LC patients (Table S3), thus emphasizing the need to perform phosphopeptide analysis.

Apart from the complex TCR signalling in the tumour microenvironment, we wish to emphasize activation of the PI3K/AKT pathway which is mediated through a range of kinases in response to insulin receptor and insulin receptor substrate signalling (Figure 6A). In LC, both kinases were upregulated four‐ to fivefold, and its aberrant regulation is frequently observed in cancers.^30^ Within this pathway, GSK3B is also threefold upregulated. A further example relates to an activation of the MAPK pathway, and in LC tumour biopsies, we measured an average fivefold increased phosphorylation of the peptide PRGQRDSSYYWEI at AS 332–344 of the RAF1 protein. Similarly, the activity of the ERK1 and ERK5 kinases increased by fivefold as evidenced by the phosphorylation of the ERK1 peptide GFLTEYVATR at AS 199–208 and the ERK5 peptide AEHQYFMTEYVAT at AS 212–224. Moreover, we observed an extraordinary induced activity of the CRK and EFS kinases, that is, 25‐ and 7‐fold, respectively. Upon MAPK activation RAF phosphorylates MEK which in turn phosphorylates ERK. Notwithstanding, an alternative route of ERK phosphorylation involves the CRK phosphorylation of the EFS kinase which in turn phosphorylates Src and eventually ERK.^31^ Interestingly, CRK selectively regulates T cell migration.^32^

### 3.3 Different kinomes in lung AD and SQ

Unlike adenocarcinomas, targeted therapy in SQ is still in its infancy.^33^ We compared the kinase activities between different histological subtypes of LC and shown in Figure 6D is the fold change difference of significantly regulated kinases between AD and SQ. We measured the activity of kinases in tumour tissue relative to NATs and compared the results between AD and SQ patients. Remarkably, of the 46 commonly regulated kinases (Figure 5B), the activities of 17 kinases are higher in SQ, and for some patients, we observed unprecedented high activities as denoted for the FGFR1, PGFRB; ANXA2, CD3ζ, GAB2, LYN and MAPK12. It was not unexpected to see higher FGFR1 activities given its common alterations in SQ. However, we also observed up to 10‐fold induced activities of the phosphoinositide‐3‐kinase regulatory subunit 1 and therefore demonstrate high activity of the phosphatidylinositol 3‐kinase subunit. Together, the data underscore the great opportunities for targeted therapies in LC, and we obtained strong evidence for a broad range of kinases which are also potential targets for the treatment of SQ.

Additionally, we compared kinase activities of patients diagnosed with driver mutations in EGFR, KRAS, p53, BRAF and MYC (Table S2) to patients without driver mutations and compile the results in Table S10. Essentially, we did not observe significant differences in kinase activities between carriers of KRAS mutations and KRAS wild type. For instance, we compared the kinase profile of > 3‐fold significantly regulated kinases of five AD patients with the KRAS mutations G13D, G12A, G12C, G12V and KRAS amplifications to nine AD patients without driver mutations and did not observe significant differences between the two cohorts (Figure 6E). The data broaden the perspective of KI‐based therapies in patients without oncogenic driver mutations.

Finally, we identified kinases which were specifically regulated in AD, and shown in Figure S7 are the signalling networks for 28 peptides. The peptides are phosphorylated by 35 different serine/threonine kinases (Table S7) and with the exception of PI3K, GSK3B, RAF and ERK, the kinases were defined by upstream analysis, that is, kinases known to phosphorylate these peptides (Figure 5D). Our findings underscore the therapeutic opportunities in blocking tumour associated signalling networks.

### 3.4 Identification of molecular hub proteins to block tumour‐specific signalling networks

**FIGURE 7:**
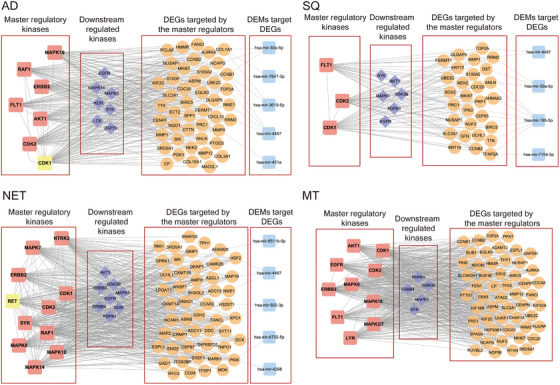
Master regulatory signalling networks in different histological types of lung adenocarcinoma. Depicted are networks of significantly upregulated kinases which function as master regulator (MR) and downstream kinase in canonical pathways. The MR augments expression of differentially expressed genes (DEGs) we found upregulated in tumours of lung cancer (LC) patients. The DEGs are targets of miRNAs which are downregulated in tumours of LC patients. Yellow coloured kinases are also upregulated at the DEG level.

We define hub proteins as master regulatory molecules which are at the apex of signalling cascades, and their inhibition results in disintegration, fragmentation and abrogation of signalling networks. By combining the kinome and genomic data, and based on computational analysis, we searched for MRs in LC‐associated signalling networks. Based on the knowledge of TRANSPATH, we obtained information on signalling molecules in different pathways and downstream reactions. We identified several kinases as MRs, and in Figure 7 we show the results for the different histological types of LC. Although the number of MRs differed between AD and SQ, that is, 7 vs 3, we did not identify specific MRs for these tumour entities. Conversely, for NET and MT, there are four (NTRK2, RET, SYK and MAPK14) and three MRs (EGFR, MAPK3 and LYN), respectively, which were specific for these histological types of LC (Figure S8). The networks shown in Figure 7 depict significantly regulated MRs, which function as kinases. Moreover, depicted are significantly downstream regulated kinases, in addition to DEGs coding for signalling molecules in the networks. Finally, miRNAs control the expression of DEGs, and for a given network, we visualize the hierarchy of signalling molecules (Figures S9–S12). The MRs function primarily as receptor tyrosine kinases, MAPKs and cyclin‐dependent kinases to influence cell proliferation, cytoskeletal dynamics and TCR/immune cell responses.

### 3.5 Validation of tumour‐regulated kinases in human LC cell lines by siRNA and CRISPER knock out and cell viability assays

We used two different publicly available data sets to validate kinases as potential therapeutic targets. First, we considered the findings of Campbell and colleagues who performed large‐scale profiling of kinase dependencies in cancer cell lines.^34^ We defined a *Z*‐score of ≥ −1.5 as threshold for significance, and only considered kinases that were significantly upregulated in > 70% of tumour samples. Among the 21 commonly upregulated kinases shown in Figure 6C, and following gene knock‐down of CDK4, FER, FES and JAK1&2, we were able to confirm loss of cell viability in 9 out of the 12 human LC cell lines. In regards to 11 kinases specifically regulated in AD (Figure 6B), siRNA‐mediated gene‐silencing of CDK1, EPHA1, JAK3, RET and ZAP70 caused loss of cell viability in a panel of six human adenocarcinoma cell lines (Table S11). Moreover, siRNA‐mediated gene silencing of the kinase ephrin receptor EPHA1 caused cell death in the large‐cell lung carcinoma cell line BEN. Concerning SQ, gene silencing of FGFR2 and PDPK1 led to significant reductions in cell viability of the human LC cell lines H460, H23 and A427. Finally, we confirmed significantly reduced cell viability in the carcinoid cell line H727 following siRNA of tyrosine kinase 2 which is specifically upregulated in carcinoid NET (Table S11).

To independently validate the relevance of regulated kinases as therapeutic targets, we interrogated the DepMap database (https://depmap.org/portal/) and retrieved kinase inhibition data for 50 human lung AD and 19 squamous carcinoma cell lines. We evaluated the impact of gene knock down on cell viability, and compared kinases significantly regulated in patient tumour samples with kinase inhibition data deposited in DepMap.^35^ Given that we compared results from different studies and experimental settings, we used −0.5 as threshold for effect size. The results are summarized in Table S11, and for each cell line, we specify the *Z*‐scores and/or effect size. Together, we confirmed 62 and 27 kinases, respectively as potential therapeutic targets in 33 human lung AD and 15 squamous carcinoma cell lines (Table S11). Therefore, by utilizing the DepMap data, we were able to validate nearly half of the kinases as potential therapeutic targets.

### 3.6 Translating multi‐omics data into individualized therapeutic concepts in distinct LC subtypes

Based on the seminal works of Hanahan and Weinberg, sustained proliferative signalling is one of the hallmark traits in cancers. In order to define kinases at the apex of signalling networks, we used kinome and genomic data and searched for master regulatory molecules as detailed in the Methods section. Depicted in Figure 7 are the MR regulatory networks. We show significantly regulated MR and downstream kinases and their associated gene networks all of which were highly regulated. We hypothesized that inhibition of MR kinases will lead to disintegration of an entire signalling trait and propose their combined inhibition with downstream kinases to be even more effective. Consequently, the gene silencing and/or drug‐dependent inhibition of the MR kinases AKT1, CDK1&2, ERBB2 and the downstream kinase EGFR markedly reduced cell viability in a large panel of human adenocarcinoma cell lines (Table S11). Similarly, gene silencing and/or drug‐dependent inhibition of CDK1&2 and the downstream kinases EGFR and MAPK1 in SQ led to marked reductions in cell viability. Noteworthy, and with the exception of AKT1 and ERBB2, estimates for the mean effect size differences ranged between −3 and −1.8. Clearly, this underscores the large effect of MR kinase inhibition on cell viability in LUAD cell lines. In regards to SQ and with the exception of MAPK1, the mean effect size differences ranged between −3.3 and −1.9. Altogether, this demonstrates the potency of MR kinase inhibition on cell viability.

By harnessing the kinase profiling data, we were able to propose new targets and/or combinations of kinase inhibitors for the treatment of LC. Specifically, of the 62 kinases upregulated in AD, and based on cell viability data following drug inhibition or gene silencing, we were able to validate 48 kinases as potential therapeutic targets across a panel of human LUAD cell lines (Table S11). We also validated 21 kinases across a panel of human lung SQ cell lines whose inhibition or gene knockdown halted or diminished cell proliferation. While some of the kinase inhibitors are already exploited for LC therapy, that is, four in AD (MET, EGFR, NTRK1 and RET) and four in SQ (EGFR, MAP2K2, RET and BRAF), there are another seven kinase targets (SRC, JAK1, TYK2, FGFR1/2, CDK6 and PDGFRA) which are used for other indications such as clear cell renal cell carcinoma, osteosarcoma, acute myeloid leukaemia, urothelial bladder cancer, bile duct and breast cancer (Table S8). We propose repurposing of these drugs and consider them as worthwhile candidates for clinical trials in LC.

**FIGURE 8:**
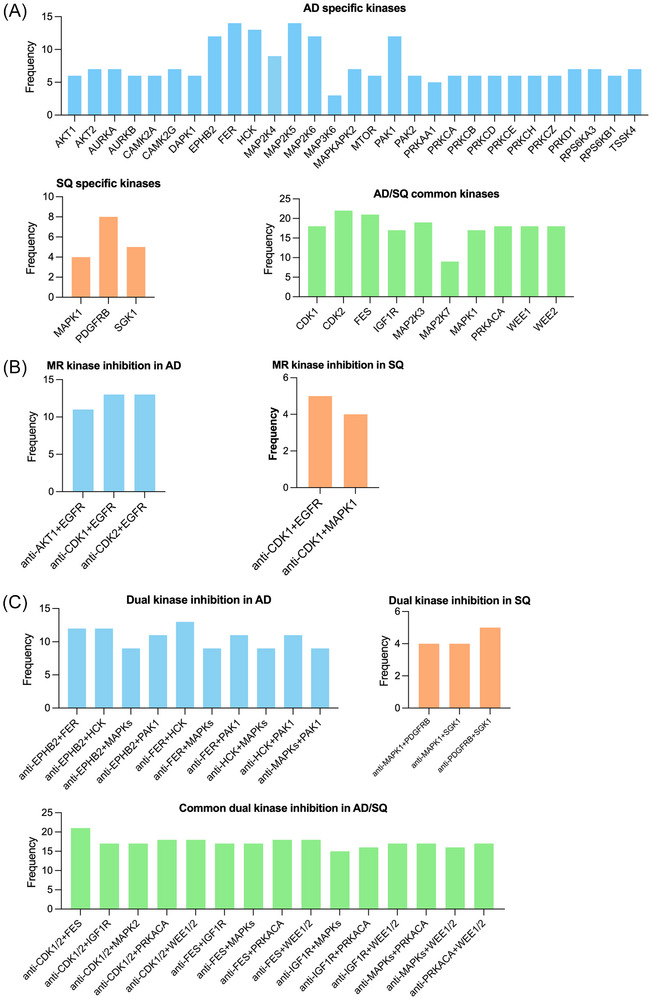
Summary of proposed targets for kinase inhibitor (KI)–based therapies in lung cancer (LC) patients. Depicted is the frequency of regulated kinases in LC patients. (A) The histogram shows the frequency of patients with kinases specifically regulated in adenocarcinoma (AD) or squamous cell carcinoma (SQ) in addition to commonly regulated in both subtypes of LC. (B) The histogram shows the frequency of patients with concurrent regulation of master regulator (MR) and downstream kinases in the same tumour sample of AD and SQ cases. We propose dual kinase inhibition to improve potency of drug treatment. (C) The histogram shows the frequency of patients with concordant regulation of kinase doublets in the same tumour sample of AD and SQ cases. Additionally, we show kinase doublets which are regulated in common in AD and SQ, and therefore these targets are independent of the LC subtypes.

Of the 62 kinases regulated in AD, there are 29 for which no approved drug is available. Similarly, for SQ, we identified three new kinases as potential therapeutic targets (Figure 8A, and Table S11). Given their significant upregulation in LC, there is good evidence for these kinases to be bona fide therapeutic targets. Some of the kinases function in cell cycle (AURKA, AURKB) or Ca2+/calmodulin dependent, mitogen‐activated protein kinases/ribosomal proteins, AKT/mTOR and various protein kinase signalling pathways as well as ephrin. Additionally, we propose PDFGRB, MAPK3 and the serine‐protein kinase Sgk1 for clinical evaluation in SQ. We also identified 10 kinases regulated in common in AD and SQ (Figure 8A).

Based on the MR networks described above (Figure 7), we hypothesized that the simultaneously use of MR and downstream kinase inhibitors will improve potency and therefore searched for LC patients with common upregulation of these therapeutic targets. The results are shown in Figure 8B, and in the case of AD, we propose the combined use of CDK1 and EGFR, CDK2 and EGFR and AKT1 and EGFR. In the case of SQ, we propose CDK1 and EGFR as well as CDK1 and MAPK1. Recently, the importance of CDK1 inhibition in cancer therapy was the subject of a review,^36^ and overexpression of EGFR is common in LC.

Apart from targeting kinases which function as MR at the apex of signalling networks, we searched for commonly regulated kinases in LC. Here, we followed the idea of dual kinase inhibition with the kinases functioning in different pathways. We set a threshold of ≥50% of patients showing upregulation of two kinases in the same tumour sample. This defined 10 kinase doublets as potential therapeutic targets (Figure 8C). Once again, the new kinase targets have been validated based on cell viability assays (Table S11) and are suitable candidates for in‐depth evaluation. In the same way, we considered kinase doublets in SQ. Outstandingly, there are 15 kinase doublets regulated in common in AD and SQ (Figure 8C).

## 4 DISCUSSION

Through comprehensive transcriptome, miRNA and kinome profiling, we examined regulatory networks in tumours of LC patients and identified 137 distinct kinases which were significantly upregulated in tumour resection material. An important finding of our study is the significant upregulation of serine/threonine kinases which are rarely exploited as drug targets in LC. Indeed, 52% of the 137 upregulated kinases are STKs. Notwithstanding, among the commonly regulated kinases (Figure 5B,D), 67% are tyrosine kinases. Based on a 2023 published update on FDA‐approved kinase inhibitors^11^ and information retrieved from the University of Dundee MRC protein phosphorylation unit (https://www.ppu.mrc.ac.uk/sites/default/files/2023‐01/small‐molecule‐inhibitors‐03‐01‐17.pdf), there are 83 kinase inhibitors targeting 24 kinases all of which were upregulated in LC (Figure 5E, blue coloured quadrants, Table S8). Moreover, of the 83 inhibitors, 20 are already in use for the treatment of LC in patients with ALK, BRAF, EGFR, ERBB2, MEK, MET, NTRK2 and RET alterations.^11^, ^40^

About 20 years ago, the landmark study of gefitinib over carboplatin‐paclitaxel revolutionized the treatment of NSCLC patients, and the IPASS study demonstrated that patients with EGFR driver mutations benefitted most from this tyrosine kinase inhibitor (TKI)–based therapy.^37^ Meanwhile, targeted therapies in LC patients with driver mutations have become the mainstay in therapy.^11^, ^38^ Yet, the selection of LC patients for a given treatment with KIs is limited to those with known driver mutations, that is, ALK, BRAF, EGFR, ERBB2, KRAS, MET, NTRK, ROS and RET alterations.^39^ Therefore, only a small set of patients benefit from KIs, and less than 10% of LC patients are considered to be eligible for KI‐based therapies.^9^ For example, targeting KRAS in LC patients carrying the KRAS G12C mutation was the subject of a recent review,^40^ and the drug sotorasib received approval in 2021 for the treatment of NSCLC. In fact, about 30% of LC patients with driver mutation are KRAS positive. Likewise, the third‐generation EGFR inhibitor osimertinib received approval for first‐line treatment in 2018, and this TKI is also effective in patients with the genotypes L858R/T790M/C797S.^41^ Gene mutations in HER2 are rare (1%–4% of NSCLC) and non‐selective TKIs are of little benefit in HER2 mutant LC patients.^42^ Notwithstanding, antibody‐drug conjugates consisting of trastuzumab‐emtansine and trastuzumab‐deruxtecan yielded impressive results regarding progression‐free survival and OS.^42^

Similarly, gene mutations in BRAF are rare among NSCLC patients (about 4%), and the importance of BRAF inhibitors in NSCLC was the subject of a recent review.^43^ In 2022, the combination of dabrafenib and trametinib for unresectable or metastatic solid tumours with BRAF V600E mutation received accelerated FDA approval. However, this combination was already approved for metastatic NSCLC in 2017. Very recently, the FDA approved the combination of encorafenib with binimetinib for BRAF mutant metastatic NSCLC.

A further example relates to the third‐generation inhibitor lorlatinib, which was approved for first‐line treatment of ALK positive NSCLC patients in 2021. Although infrequent, ALK gene fusions are more common in NSCLC when compared to gene mutations and gene amplifications. Finally, the results of the Geometry Mono‐1 study (NCT02414139) in exon 14 skipping and MET amplified NSCLC patients was published in 2020,^44^ and the impressive capmatinib antitumour activity led to its approval in 2022.

Collectively, the benefit of kinase inhibitors in LC are unquestionable but until now such therapies are narrowly focused on patients with driver mutations. Given that 21 kinases are regulated in LC irrespective of the histological subtypes, we propose a much broader use of kinase inhibitors (Figure 6C). In addition, we broaden the perspective of STK‐based treatment algorithms in LC and identified 35 STKs specifically regulated in AD (Figure 5D) of which CREB1 is a prominent example.

Furthermore, the results of combined immune checkpoint and kinase inhibitor therapy was recently summarized,^45^ and a triple therapy consisting of atezolizumab, the BRAF inhibitor vemurafenib and the MEK inhibitor cobimetinib was approved as first‐line treatment in BRAFV600 melanoma patients. It therefore can be regarded as a proof‐of‐concept study. Notwithstanding, the combination of ICIs with TKIs in LC patients with EGFR mutations is disappointing and is associated with significant toxicities especially in the combination pembrolizumab with gefitinib.^46^ Although the combination of pembrolizumab with erlotinib is feasible, it is not superior to erlotinib monotherapy as observed in the ClinicalTrials.gov ID NCT02039674; A Study of Pembrolizumab (MK‐3475) in Combination With Chemotherapy or Immunotherapy in Participants With Non‐small Cell Lung Cancer (MK‐3475‐021/KEYNOTE‐021) sponsored by Merck Sharp & Dohme LLC. Obviously, new studies are needed to evaluate efficacy and safety of combination therapies (Table 1).

**TABLE 1:** Patient characteristics.

Characteristic	Adenocarcinoma (*N* = 54)	Squamous cell carcinoma (*N* = 23)	Neuroendocrine tumour (*N* = 9)	Metastatic tumour (*N* = 9)
Age, years				
Median	71.5	74	73	59
Range	28–91	49–87	57–82	23–82
Sex				
Female	22 (40.7%)	9 (39.1%)	4 (44.4%)	4 (44.4%)
Male	32 (59.3%)	14 (60.9%)	5 (55.6%)	5 (55.6%)
Smoking status				
Never smoker	24 (44.4%)	8 (34.8%)	4 (44.4%)	4 (44.4%)
Former smoker	5 (9.3%)	1 (4.3%)	1 (11.1%)	0 (0%)
Current smoker	22 (40.7%)	14 (60.9%)	4 (44.4%)	2 (22.2%)
N/A	3 (5.6%)	0 (0%)	0 (0%)	3 (33.3%)
TNM stage at diagnosis				
I–II	38 (70.4%)	11 (47.8%)	5 (55.6%)	0 (0%)
IIIA	7 (13.0%)	9 (39.1%)	3 (33.3%)	0 (0%)
IIIB	2 (3.7%)	1 (4.3%)	0	0 (0%)
IV	2 (3.7%)	0 (0%)	0	9 (100%)
N/A	5 (9.3%)	2 (8.7%)	1 (11.1%)	0 (0%)
Treatment prior to surgery				
Yes	0 (0%)	3 (13.0%)	0 (0%)	100 (100%)
No	100 (100%)	20 (87%)	100 (100%)	0 (0%)
EGFR mutation				
Yes	1 (1.8%)	0 (0%)	0 (0%)	0 (0%)
No	22 (40.7%)	4 (17.4%)	1 (11.1%)	0 (0%)
N/A	31 (57.4%)	19 (82.6%)	8 (88.9%)	100 (100%)
TP53 mutation				
Yes	8 (14.8%)	3 (13.0%)	1 (11.1%)	0 (0%)
No	15 (27.8%)	1 (4.3%)	0 (0%)	0 (0%)
N/A	31 (57.4%)	19 (82.6%)	8 (88.9%)	100 (100%)
KRAS mutation/amplification				
Yes	16 (29.6%)	1 (4.3%)	0 (0%)	0 (0%)
No	7 (13.0%)	3 (13.0%)	1 (11.1%)	0 (0%)
N/A	31 (57.4%)	19 (82.6%)	8 (88.9%)	100 (100%)
PD‐1 expression				
<1%	17(31.5%)	16 (69.6%)	2 (22.2%)	2 (22.2%)
≥1%	29 (53.7%)	3 (13.0%)	2 (22.2%)	0 (0%)
N/A	8 (14.8%)	4 (17.4%)	5 (55.6%)	7 (77.8%)

Abbreviations: EGFR, epidermal growth factor receptor; KRAS, Kirsten rat sarcoma virus; TNM, Tumour Node Metastasis.

Based on the concept of MR networks, we used multi‐omics data to define master regulatory networks with kinases functioning at the apex of signalling networks. We propose their inhibition as a novel therapeutic concept and identified combinations whereby the MR and downstream kinases are concomitantly inhibited as shown in Figure 8B. Moreover, we identified 10 kinase doublets as potential therapeutic targets and propose their inhibition to be more potent than single kinase inhibition.

While the use of TKIs in LC treatment reduced significantly cancer‐related mortality, the risk of adverse cardiac and cerebrovascular events is significant, as reported for a large cohort of > 24 000 patients on TKI treatment which was compared to an equal large cohort of > 24 000 patients not receiving TKIs.^47^ Apart from cardiovascular adverse effects, that is, hypertension, atrial fibrillation, impaired cardiac function and even heart failure, common ADRs for gefitinib include skin toxicity and diarrhoea as well as nausea, interstitial lung disease, dry skin, pruritus, stomatitis and anorexia. In a recent review, the spectrum of adverse effects of TKI‐based cancer therapy was summarized, and as denoted by the authors, the potential mechanisms are mostly unclear thus leaving a critical knowledge gap.^48^

Furthermore, most, if not all patients on targeted therapies develop drug resistance which render TKI ineffective,^49^ and possible reasons are modifications of the oncogenic driver itself, changes in the drug target expression, activation of parallel and alternate signalling pathways, activation of downstream signalling and histological changes such as epithelial‐to‐mesenchymal transition. The various concepts for drug resistance to TKI based therapies have been summarized.^49^, ^50^

**FIGURE 9:**
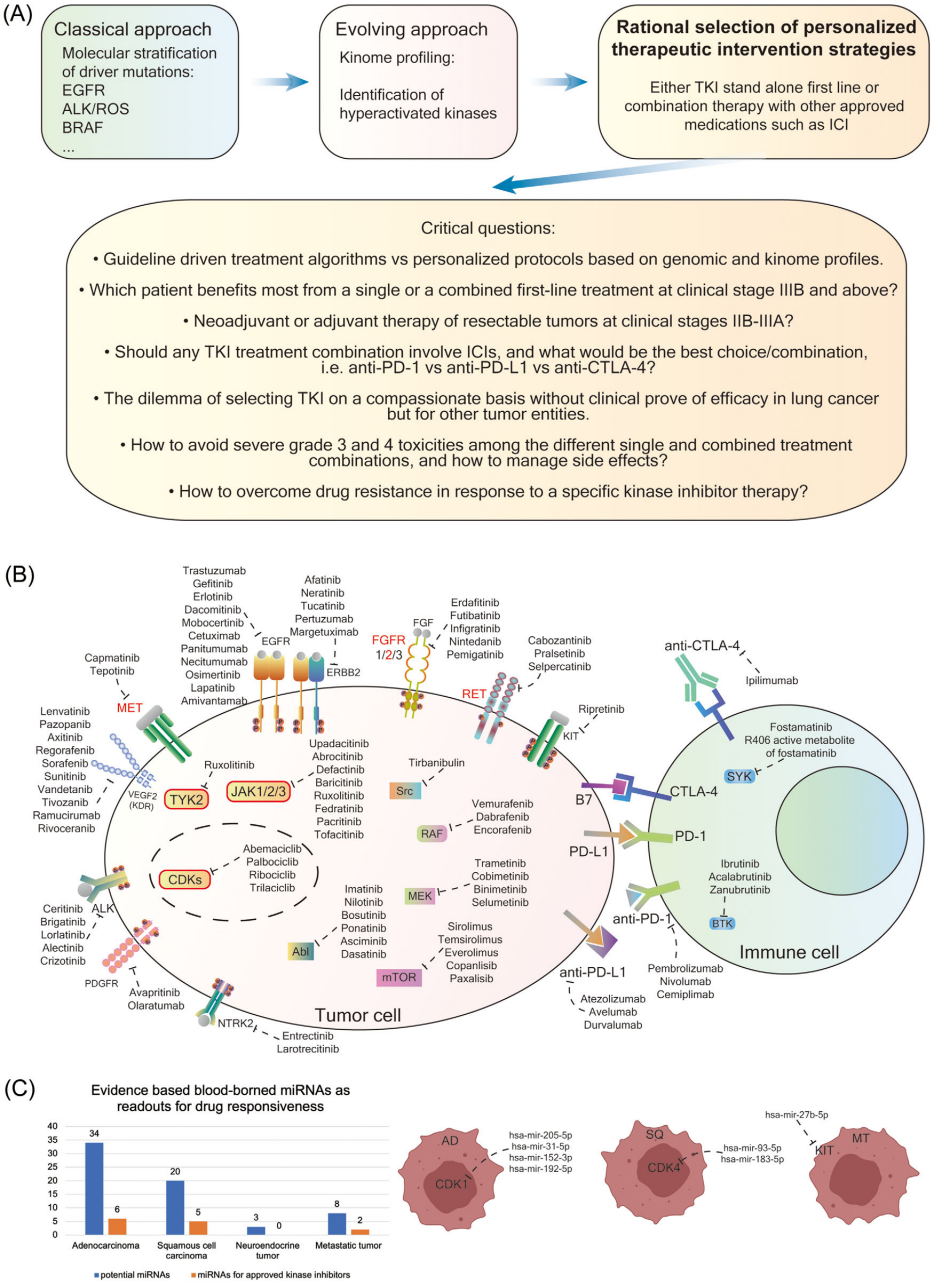
The landscape of kinase targets in lung cancer (LC) and immune cells. (A) Depicted is the current and evolving paradigm of tyrosine kinase inhibitor (TKI)‐based therapies, and the critical questions that need to be considered. (B) Overview of drugs targeting kinases in lung cancer and other solid tumours. (C) Histogram of the number of blood borne miRNAs that have the potential to serve as biomarker for safety and efficacy of kinase‐based therapies and examples of specifically regulated miRNAs in adenocarcinoma (AD), squamous cell carcinoma (SQ) and metastatic tumours (MT) that respond to CDK1, CDK4 and KIT inhibitors.

Together, the combined use of kinome and genomic data enabled us to construct regulatory networks. Targeting MR hub proteins and downstream kinases is a novel approach and permits the development of a personalized therapeutic concept based on aberrant signalling networks which are independent of driver mutations. Potentially, new concepts for overcoming drug resistance emerge through combined inhibition of MR‐ and downstream kinases. In Figure 9A, we show the current and evolving concepts in TKI‐based therapies, and we highlight the classical and evolving approach that combines genetic, genomic and kinome data.

Although we identified the remarkable regulation of > 40 kinases in the majority of patients (Figure S6f), the following caveats and critical questions need to be considered:

1. Are only patients with advanced LC tumours, that is, unresectable Stage III, eligible for the proposed treatment algorithm?
2. The difficulties to select drugs and doses for personalized treatments based on the kinome profile that have no approval for LC but other tumour indications.
3. Acceptance for personalized treatment regimens when they deviate from guideline driven treatment algorithms.
4. How to evaluate safety and efficacy in the absence of data from randomized blinded clinical trials? In other words, do we need to develop a new conceptional framework for an assessment of evidence from personalized treatment and the collection and reporting of such findings that in its cumulation would be an equivalent to randomized clinical trials?
5. Uncertainties regarding efficacy and safety of mono over combination therapies with other kinase inhibitors, ICIs and other therapeutics.
6. How to avoid severe toxicities in the various treatment algorithms, and how to manage them?

Depicted in Figure 9B are the various targets which are currently exploited in the treatment of LC and other solid tumours. Furthermore, we identified blood borne DEMs, which control expression of highly regulated kinase in LC, and Figure 9C shows the wealth of blood borne miRNAs that carry the potential to serve as biomarker for safety and efficacy of kinase‐based therapies. We also provide examples for specific blood borne miRNAs linked to CDK inhibitors and the receptor kinase KIT. Thus, we raise the possibility of considering DEMs as predictive biomarkers for KI treatment. Depicted in the graphical abstract is a schema of the proposed therapeutic concept, and the underlying decision tree for the selection of kinase inhibitors.

## 5 CONCLUSIONS

We propose the broad use of kinase inhibitors based on the individual needs of a LC patient.

## AUTHOR CONTRIBUTIONS

*Experimental investigations*: Shen Zhong and Yvonne Börgeling. *Data curation and analysis*: Shen Zhong. *Visualization*: Shen Zhong. *Bioinformatics*: Shen Zhong. *Data interpretation*: Shen Zhong. *Extended literature search*: Shen Zhong and Jürgen Borlak. *Treating physician*: Patrick Zardo. *Surgical removal of the tumour*: Patrick Zardo. *Clinical data collection*: Patrick Zardo. *Funding acquisition*: Patrick Zardo and Jürgen Borlak. *Pathological classification of tumours*: Danny Jonigk. *Conceptualization*: Jürgen Borlak. *Data analysis and interpretation*: Jürgen Borlak. *Supervision*: Jürgen Borlak. *Manuscript preparation*: Jürgen Borlak.

## CONFLICT OF INTEREST STATEMENT

The authors declare no conflicts of interest.

## ETHICS STATEMENT

The ethics committee of Hannover Medical School approved the study (3381‐2016), and we obtained informed consent from all patients for the use of tissue resection material who underwent surgery for lung cancer at Hannover Medical School during the period 2017–2022.

## CONSENT FOR PUBLICATION

All authors have read the manuscript and approved for publication.

## Supporting information



Supporting Information

Supporting Information

Supporting Information

Supporting Information

Supporting Information

Supporting Information

Supporting Information

Supporting Information

Supporting Information

Supporting Information

Supporting Information

Supporting Information

## Data Availability

The data are available in the Supporting Information, and the entire kinome and genomic data will be made available through public repositories.
