# Supplementary material for: Comprehensive transcriptome, miRNA and kinome profiling identifies new treatment options for personalized lung cancer therapy
Source: Clin Transl Med. 2025 Feb 24;15(3):e70177. doi: 10.1002/ctm2.70177 (PMC11850761; doi:10.1002/ctm2.70177)

Supplementary Fig. S1. Examples of 18S and 28S ribosomal bands on denaturing agarose gels.

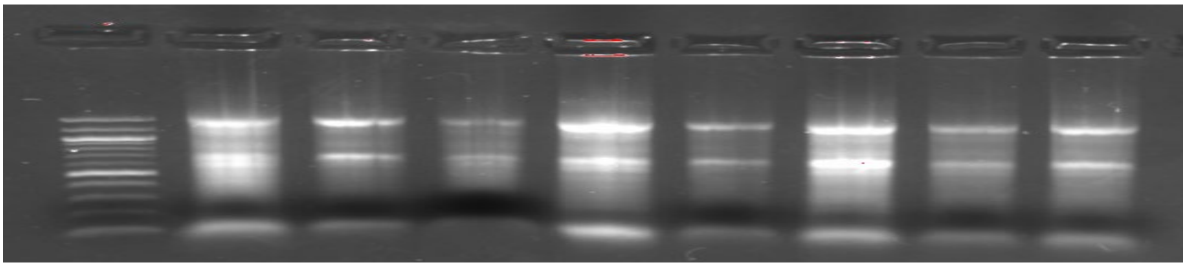

Supplementary Fig. S2. PCA and heatmaps of the genomic profiling.

Panel A: PCA analysis of the gene profiling (i-iv) and miRNA profiling (v-viii) of lung adenocarcinoma, squamous cell carcinoma, neuroendocrine tumors and metastatic tumors. Blue colored dots are non-tumorous adjacent tissue; red colored dots are tumors.

Panel B: Heatmaps of the significantly regulated genes (i-iv) and miRNAs (v-viii) in lung adenocarcinoma, squamous cell carcinoma, neuroendocrine tumors and metastatic tumors. The tumor associated DEGs are clearly separated from non-tumorous adjacent tissue.

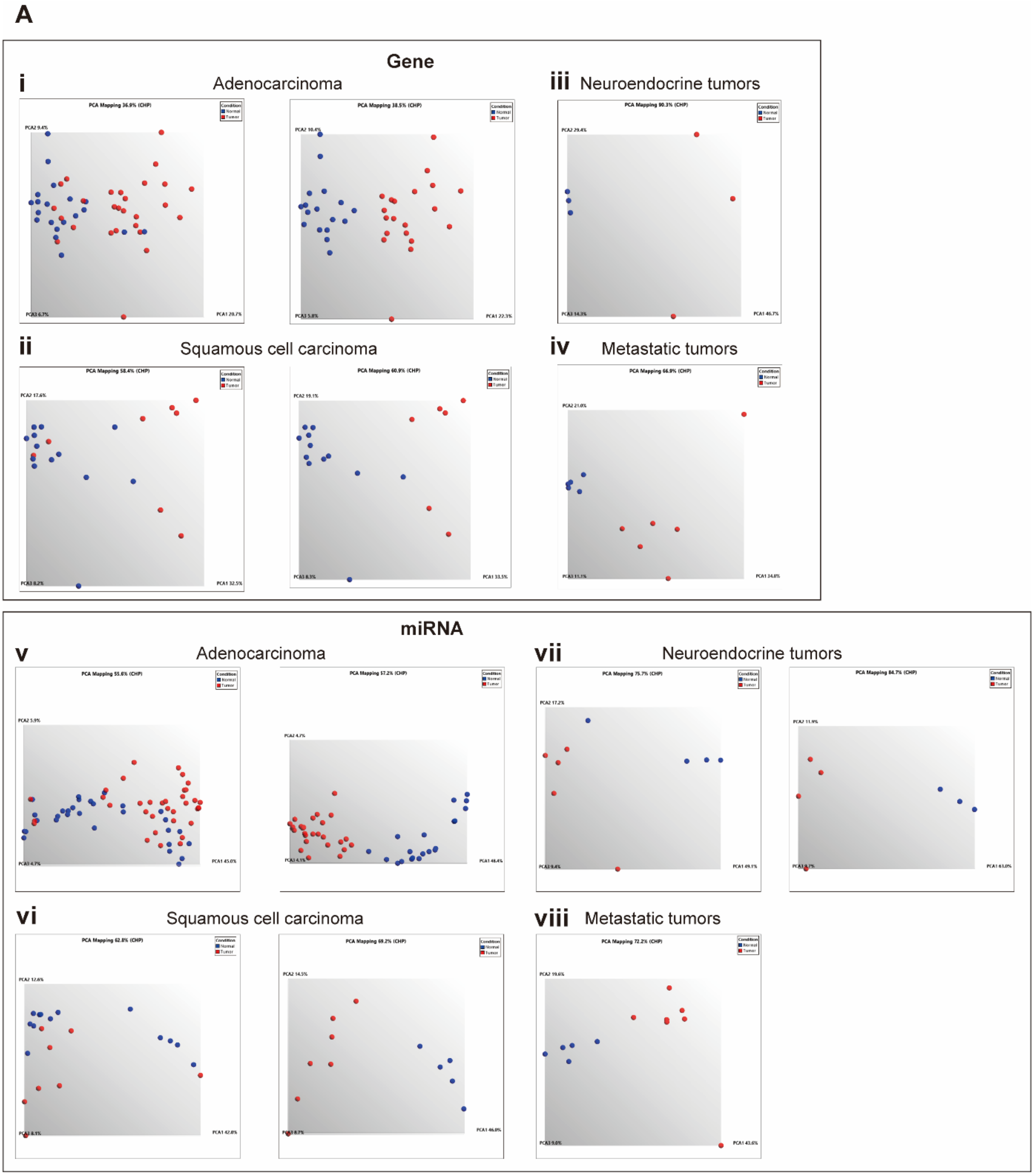

B

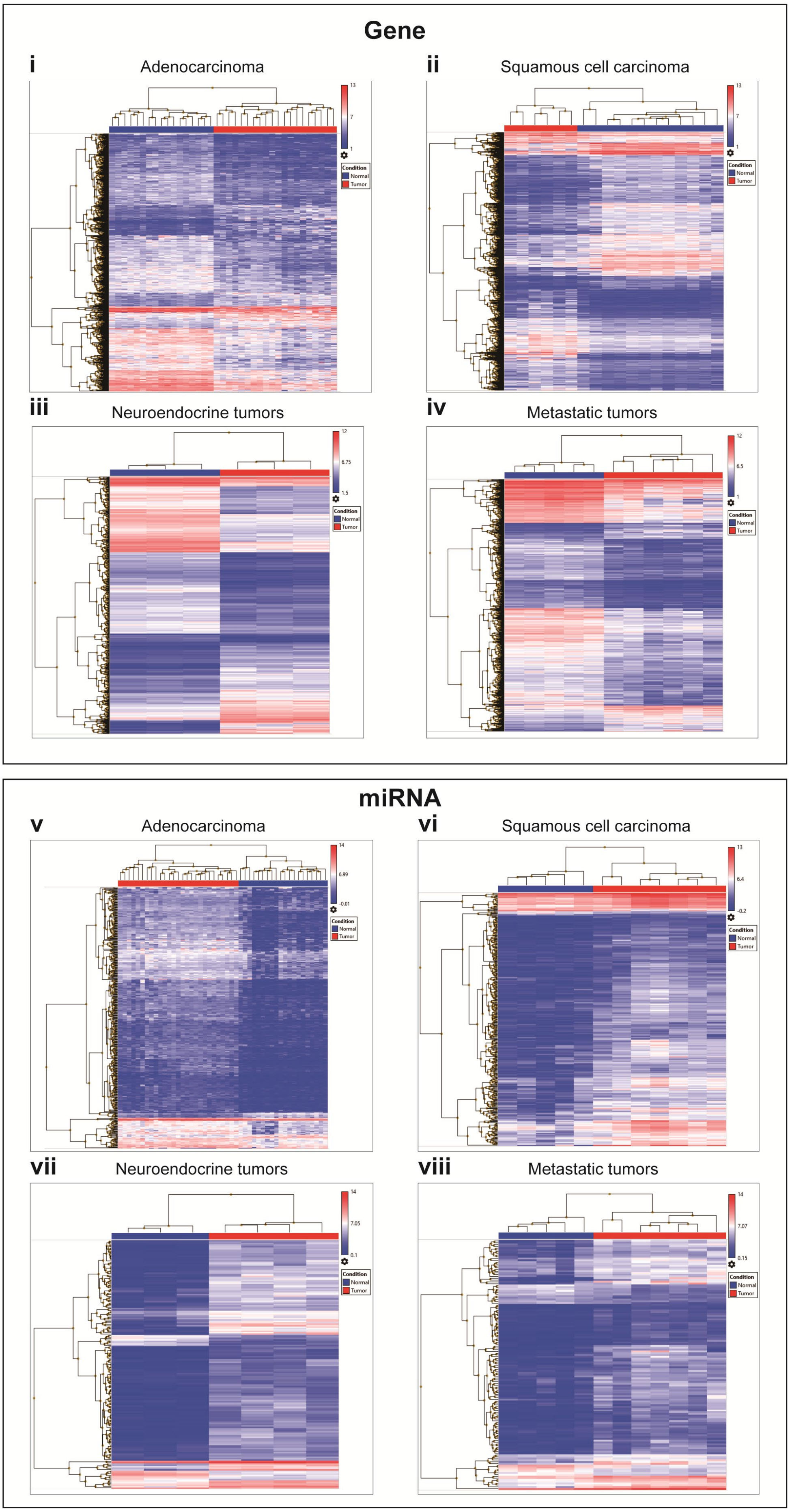
Gene
i
Adenocarcinoma
ii
Squamous cell carcinoma
iii
Neuroendocrine tumors
iv
Metastatic tumors
miRNA
v
Adenocarcinoma
vi
Squamous cell carcinoma
vii
Neuroendocrine tumors
viii
Metastatic tumors

Supplementary Fig. S3. Venn diagrams of the significantly enriched GO terms of DEGs specific for AD, SC, NET and MT.

AD: adenocarcinoma; SC: squamous cell carcinoma; NET: neuroendocrine tumors; MT: metastatic tumors.

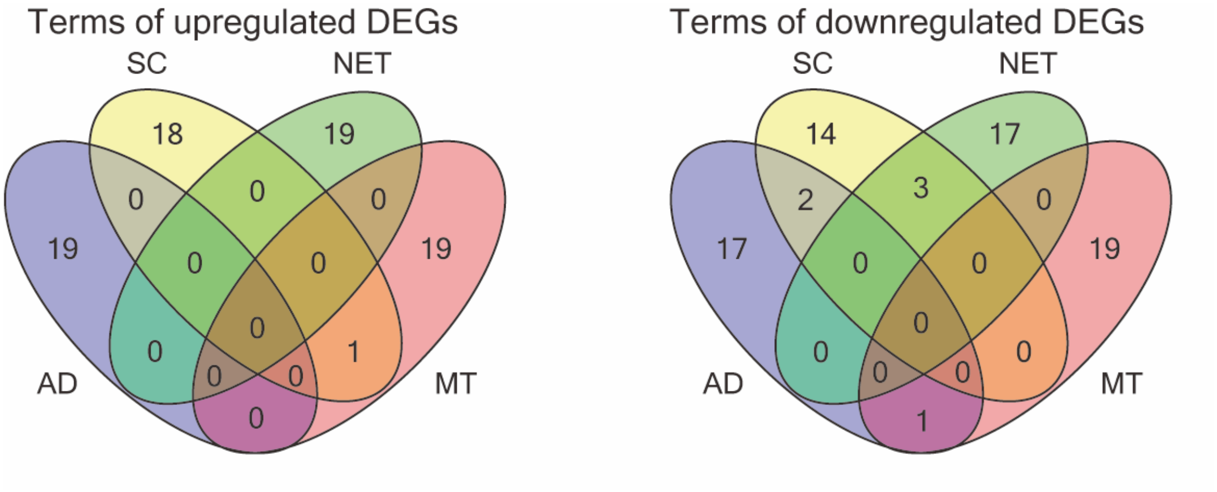

Supplementary Fig. S4. Example of kinetic plots for tyrosine kinases in AD and SC tumors and matched non-tumorous adjacent tissue.

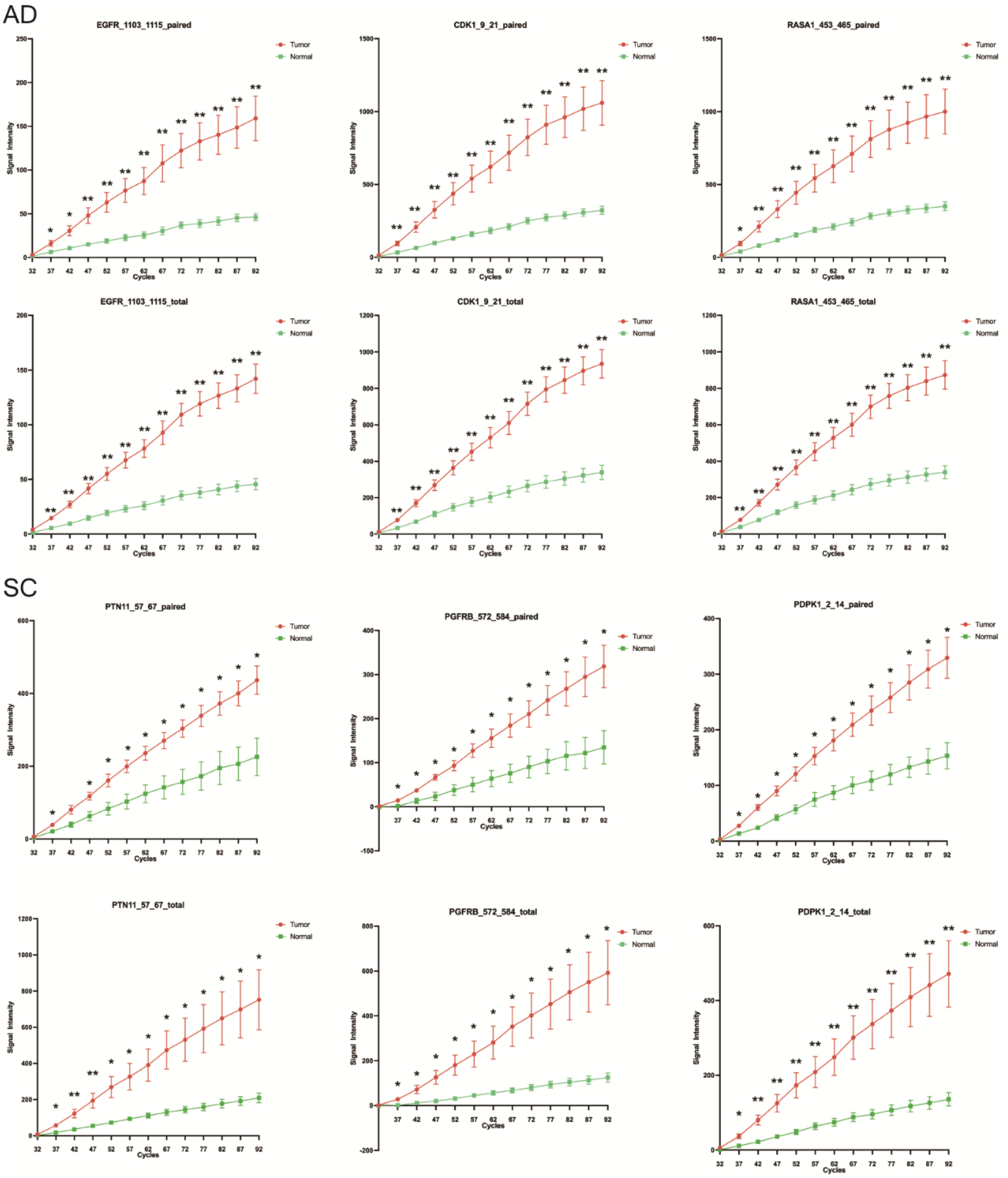

Supplementary Fig. S5. Venn diagrams of the significantly regulated miRNAs and the multi query types and additional kinases from correlation analysis.

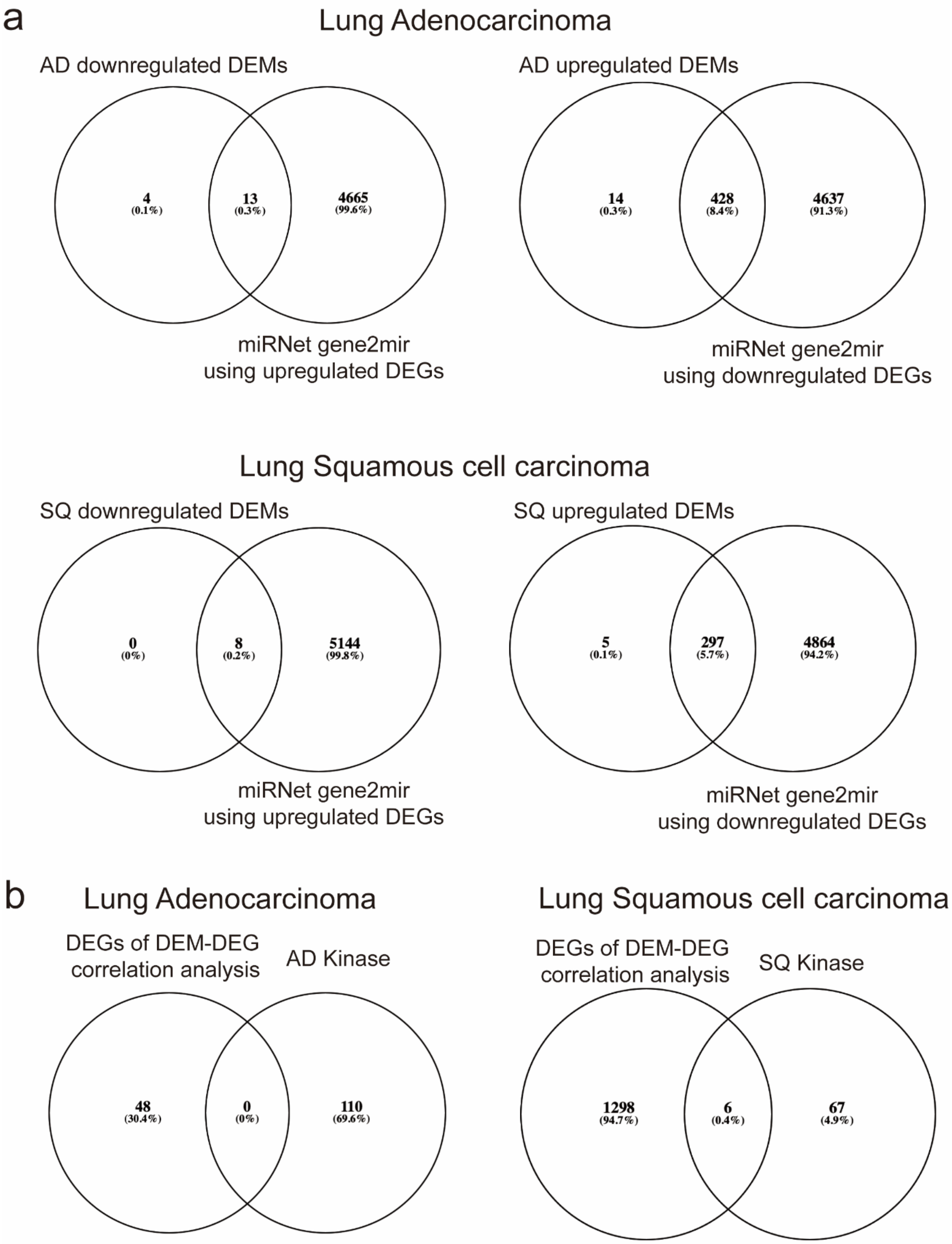

Supplementary Fig. S6: Upstream kinase analysis.

Panel a-d: Kinome trees of AD, SC, NET and MT based on upstream kinase analysis.

Panel e: Venn diagram of regulated kinases based on direct measurement and upstream kinases analysis.

Panel f: Histogram of the number of kinases regulated in lung cancer patients.

Panel g: Venn diagram of significantly regulated kinases in the various types of lung cancer.

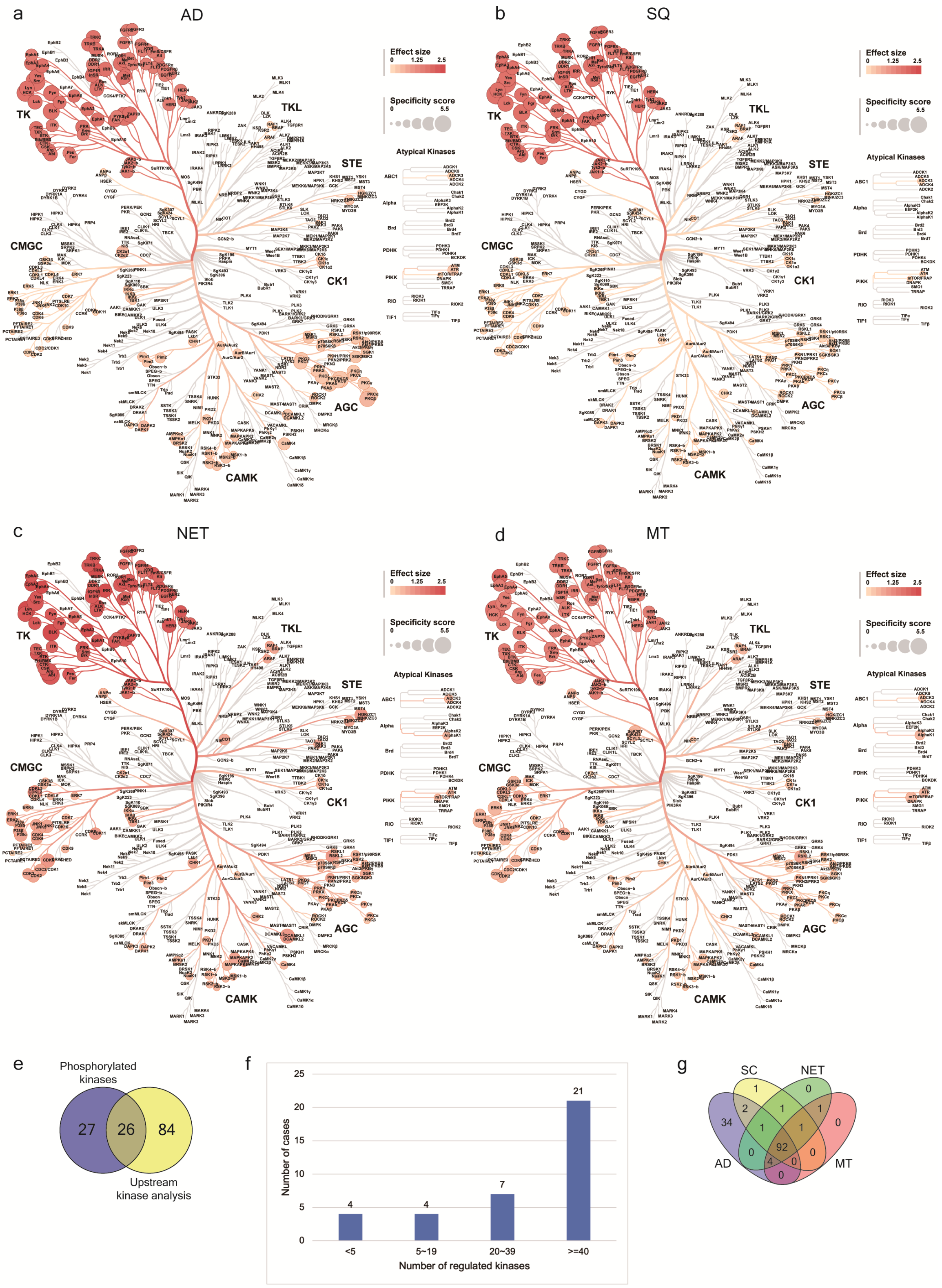

Supplementary Fig. S7. Signaling networks of AD specific kinases.

Panel a: Dot plots of regulated kinases between lung adenocarcinoma and squamous cell carcinoma. The data are fold changes and statistical testing is based on a two-sided t test when the data are normally distributed; otherwise, we used the two-sided Mann-Whitney U test. Error bars represent 95% confidence intervals.

Panel b: Signaling networks of serine-threonine kinases specifically regulated in lung adenocarcinoma. Kinases marked with ℗ represent direct phosphorylation of the protein. Rectangular shaped kinases are based on upstream kinase analysis. Grey colored kinases were not measured. Note, PI3K, RAF, MEK, ERK and GSK3B are important kinases in the signaling pathways and are commonly regulated in AD, SC, NET and MT.

a

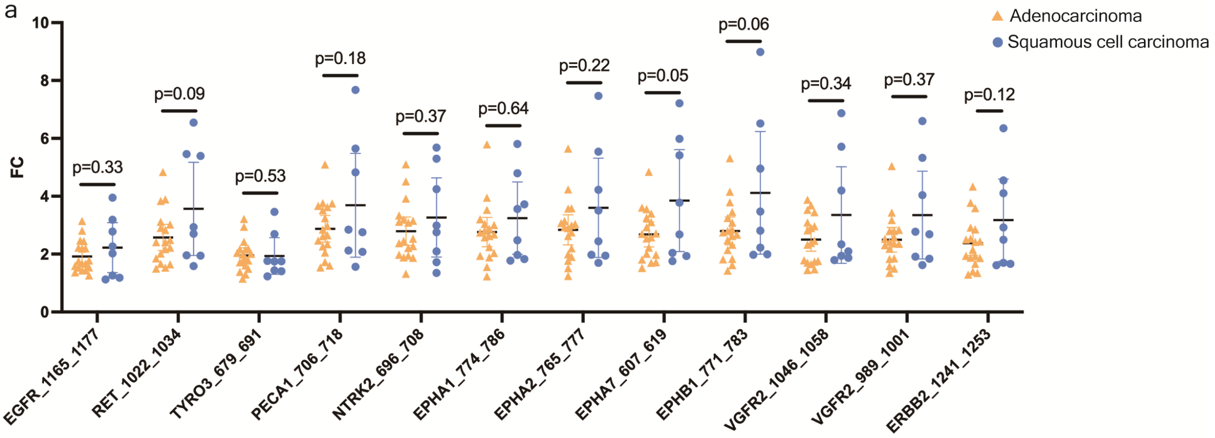

b

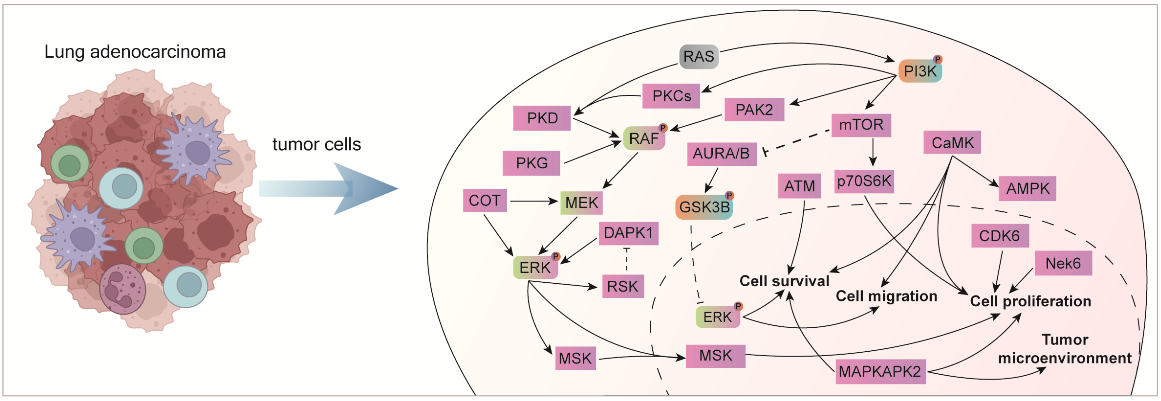

Supplementary Fig. S8. Venn diagram of master regulators identified in the various types of lung cancer.

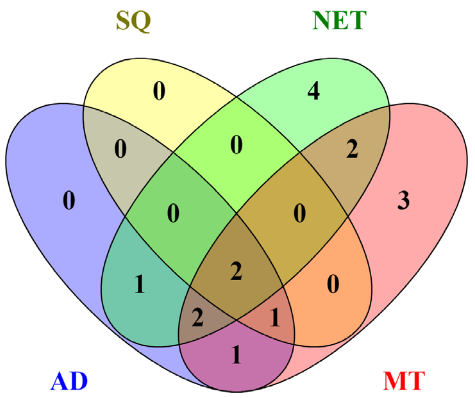

Supplementary Fig. S9. Master regulatory network of lung adenocarcinoma.

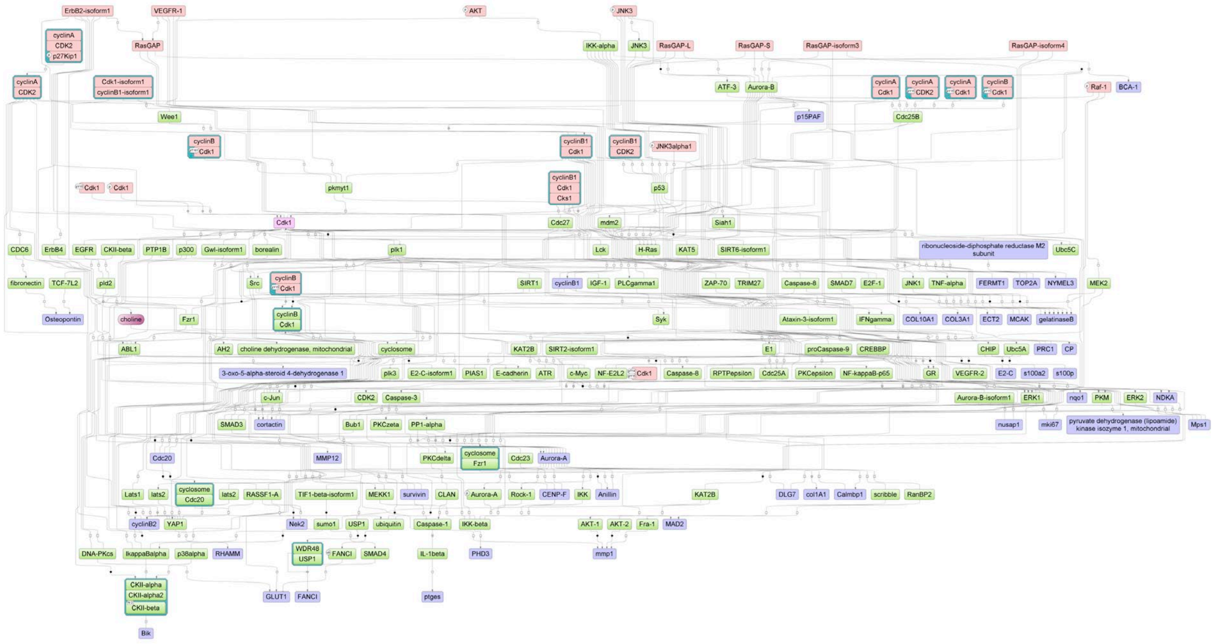

Supplementary Fig. S10. Master regulatory network of lung squamous cell carcinoma. Pink color represents the master regulators. Purple color represents the significantly regulated genes. Green color represents the molecules in the signaling pathway but are not significantly regulated at the DEG level.

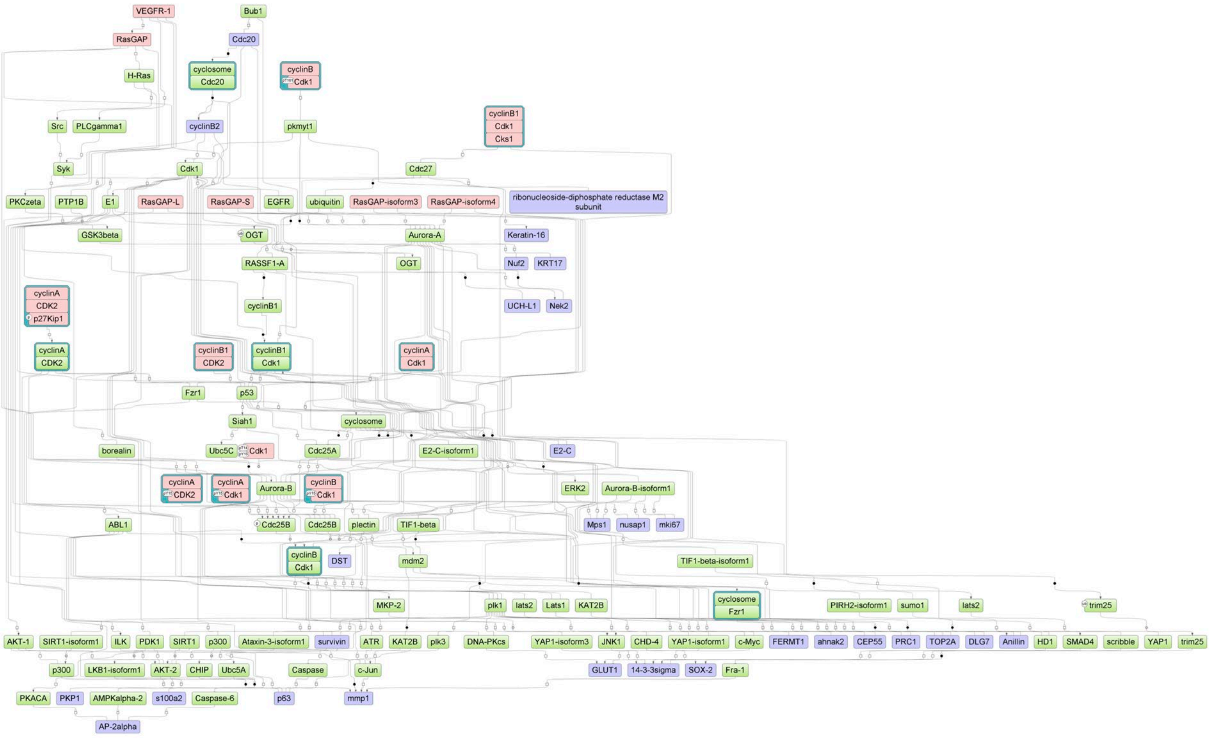

Supplementary Fig. S11. Master regulatory network of neuroendocrine tumors. Pink color represents the master regulators. Purple color represents the significantly regulated genes. Green color represents the molecules in the signaling pathway but are not significantly regulated at the DEG level.

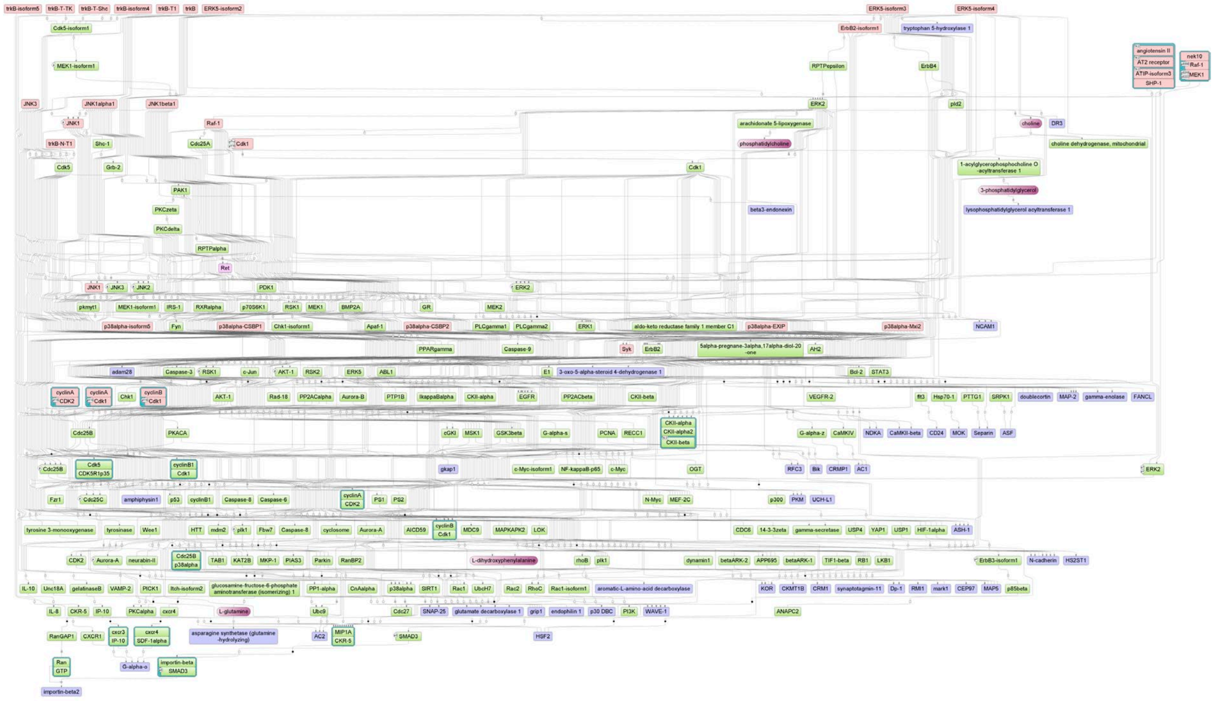

Supplementary Fig. S12. Master regulatory network of metastatic tumors. Pink color represents the master regulators. Purple color represents the significantly regulated genes. Green color represents the molecules in the signaling pathway but are not significantly regulated at the DEG level.

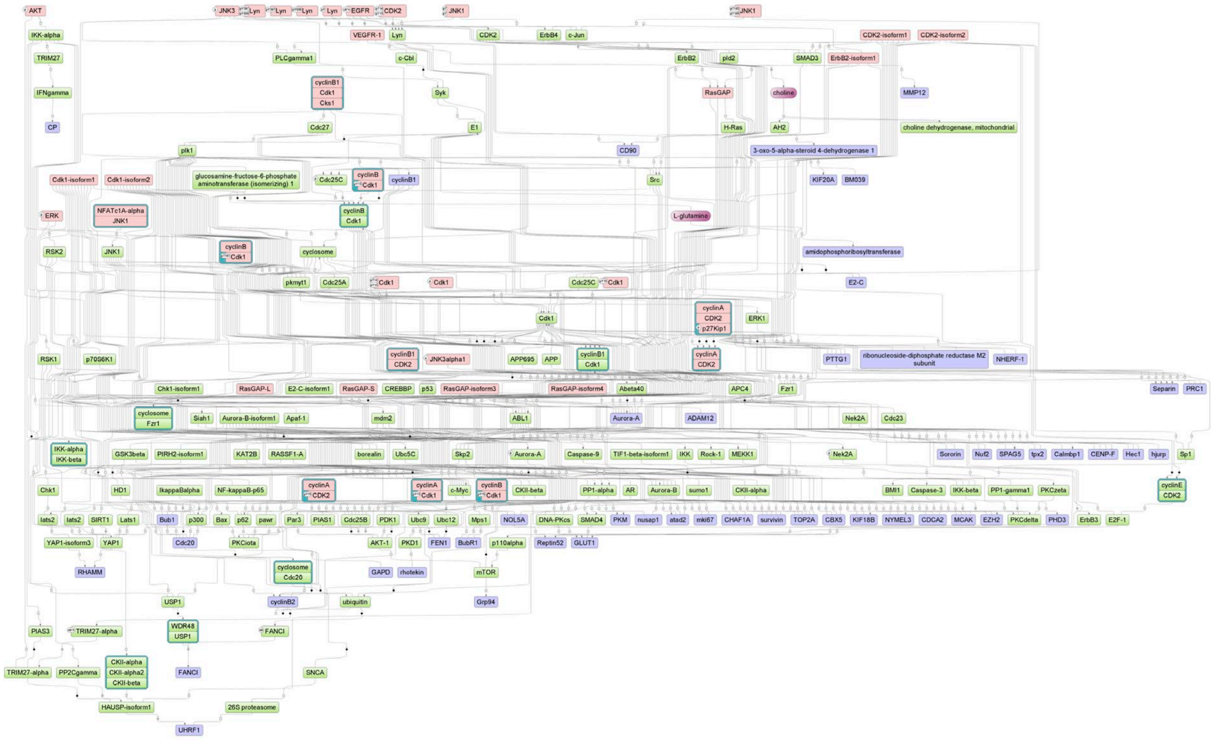

Supplement: Supplementary file 1 — Supporting Information [file CTM2-15-e70177-s009.pdf]
