# Supplementary material for: Comprehensive transcriptome, miRNA and kinome profiling identifies new treatment options for personalized lung cancer therapy
Source: Clin Transl Med. 2025 Feb 24;15(3):e70177. doi: 10.1002/ctm2.70177 (PMC11850761; doi:10.1002/ctm2.70177)
Supplement: Supplementary file 2 — Supporting Information [file CTM2-15-e70177-s012.docx]

|  | Whole genome gene profiling | | Whole genome miRNA profiling | | Kinome profiling | |
| --- | --- | --- | --- | --- | --- | --- |
| Type of tumour | Total | After PCA | Total | After PCA | Total | After PCA |
| AD | 32 | 28 | 43 | 39 | 31 | 29 |
| SQ | 14 | 13 | 14 | 11 | 17 | 11 |
| NET | 4 | 4 | 6 | 6 | 6 | 6 |
| MT | 6 | 6 | 7 | 7 | 8 | 8 |

Supplementary Table S1: Clinicopathological information of lung cancer patients.
